# Noncovalent Interactions with PAMAM and PPI Dendrimers Promote the Cellular Uptake and Photodynamic Activity of Rose Bengal: The Role of the Dendrimer Structure

**DOI:** 10.1021/acs.jmedchem.1c01080

**Published:** 2021-09-21

**Authors:** Krzysztof Sztandera, Michał Gorzkiewicz, Ana Sofia Dias Martins, Lorenzo Pallante, Eric Adriano Zizzi, Marcello Miceli, Mateusz Ba̧tal, Catarina Pinto Reis, Marco A. Deriu, Barbara Klajnert-Maculewicz

**Affiliations:** † Department of General Biophysics, Faculty of Biology and Environmental Protection, University of Lodz, 141/143 Pomorska St., 90-236 Lodz, Poland; ‡ iMed.ULisboa−Research Institute for Medicines, Faculdade de Farmácia, Universidade de Lisboa, Av. Prof. Gama Pinto, 1649-003 Lisboa, Portugal; § Polito^BIO^MedLab, Department of Mechanical and Aerospace Engineering, Politecnico di Torino, Corso Duca degli Abruzzi 24, 10129 Turin, Italy; ∥ Instituto de Biofísica e Engenharia Biomédica, Faculdade de Ciências, Universidade de Lisboa, Campo Grande, 1749-016 Lisboa, Portugal

## Abstract

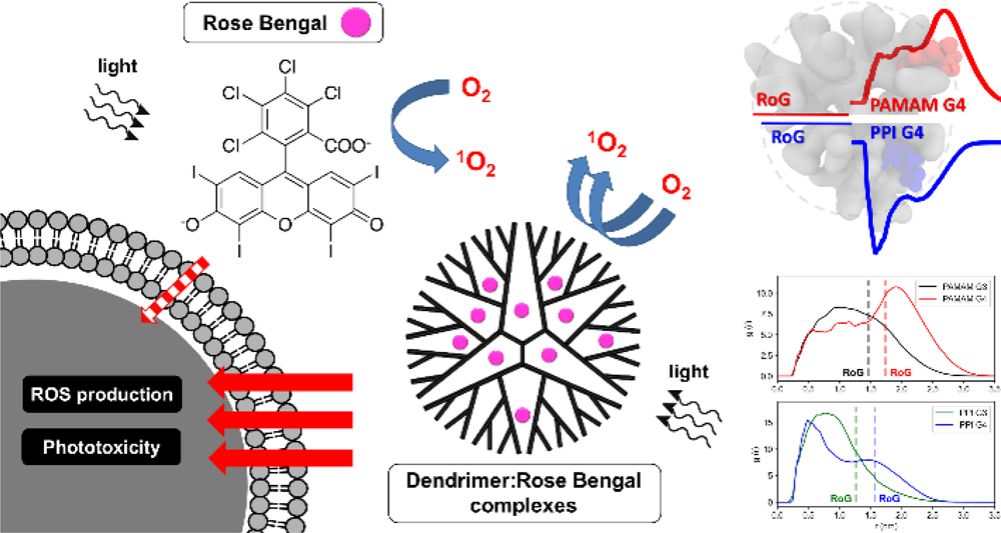

Rose bengal is an anionic dye considered as a potential photosensitizer for anticancer photodynamic therapy. The clinical utility of rose bengal is hampered by its short half-life, limited transmembrane transport, aggregation, and self-quenching; consequently, efficient drug carriers that overcome these obstacles are urgently required. In this study, we performed multilevel *in vitro* and *in silico* characterization of interactions between rose bengal and cationic poly(amidoamine) (PAMAM) and poly(propyleneimine) (PPI) dendrimers of the third and fourth generation and assessed the ability of the resultant complexes to modulate the photosensitizing properties of the drug. We focused on explaining the molecular basis of this phenomenon and proved that the generation- and structure-dependent binding of the dye by the dendrimers increases the cellular uptake and production of singlet oxygen and intracellular reactive oxygen species, leading to an increase in phototoxicity. We conclude that the application of dendrimer carriers could enable the design of efficient photodynamic therapies based on rose bengal.

## 1 Introduction

Photodynamic therapy (PDT) is one of the most promising methods for the treatment of basal cell carcinoma and different types of skin cancer.^1^ This highly specific approach is primarily based on the application of a light-sensitive compound (so-called photosensitizer, PS), which, upon excitation with light of a certain wavelength, generates reactive oxygen species (ROS). This, in turn, leads to the oxidation of cellular nucleic acids, lipids, and proteins, disrupting cell signaling cascades or gene regulation and ultimately activating several cell death pathways.^2^ Such a specific mechanism enables treatment to be targeted precisely to the area of a neoplastic lesion upon direct application of PS and light.^3^ Thus, the benefits of PDT are its noninvasiveness and lack of adverse side effects. However, the level of damage and the mechanisms of cell death depend not only on the clinical setup (e.g., time of irradiation and light intensity) but also on the properties, concentration, and subcellular localization of PS.^4^ Consequently, to take full advantage of the potential of PDT, it is essential to select the appropriate phototoxic drug.

The ideal PS should have the following properties: maximum absorbance between 650 and 850 nm, high efficiency of free radical production, low photodegradation, and nontoxicity in the dark. Additionally, PSs should have long half-lives and efficient cellular uptake, enabling sufficient intracellular accumulation to trigger a toxic effect.^4,5^ Despite many years of research, clinically used PSs remain far from perfect.

Rose bengal (4,5,6,7-tetrachloro-2′,4′,5′,7′-tetraiodofluorescein; RB) is a dianionic fluorescent dye belonging to the class of xanthenes. RB is currently approved as an ocular diagnostic tool and has been designated by the Food and Drug Administration (FDA) for the treatment of several types of cancers and skin conditions.^5^ Due to its high efficiency of singlet oxygen generation,^6^ RB is considered a good candidate to serve as a PS in anticancer PDT. However, the potential use of RB in the photodynamic therapy of neoplasms is limited mainly by its short half-life, hydrophilic nature, and tendency to aggregate. RB is negatively charged at physiological pH, hindering transmembrane transport and preventing the accumulation of clinically relevant intracellular concentrations. Its half-life (∼30 min) further limits distribution and tissue accumulation; consequently, multiple dosing may be needed to reach the optimal therapeutic effect. In addition, RB forms aggregates in solutions, which affect the spectral properties of the dye and cause a decrease in its photodynamic activity, including the ability to generate singlet oxygen and other ROS.^5^

To overcome the limitations associated with photo-instability, poor biodistribution, and cellular uptake, the use of the appropriate RB formulation or delivery system may be a promising approach. Clinically used lipidic and organic formulations of PSs may yield unpredictable distribution patterns, allergic reactions, hypersensitivity, and systemic toxicity.^7^ To overcome these problems, researchers have turned to the field of nanotechnology, which has the potential to generate nanoscale particles with precisely defined features.^8,9^ Here, dendrimers are a class of nanoparticles that has been studied comprehensively both *in vitro* and *in vivo* in the context of anticancer drug delivery.^10−12^ These sphere-shaped, water-soluble polymers of symmetrical, well-defined structure protect drugs from degradation, extend their half-life, promote intracellular transport,^13^ and provide semispecific accumulation in tumor regions; the latter phenomenon is referred to as the enhanced permeability and retention (EPR) effect.^14^

The three-dimensional architecture and chemical composition of dendrimers offer several options for the attachment of drugs. In particular, therapeutics can be physically entrapped inside the dendritic scaffold or linked by noncovalent interactions or covalent bonds, both on the surface and within the dendrimer structure.^15^ In the context of PDT, an additional advantage is that optimized release of PS from the carrier at the target site is not required for the cytotoxic effect so long as the nanocarrier does not limit the diffusion of molecular oxygen.^8^ However, although dendrimer/drug conjugates are generally more stable in solutions and *in vivo*, the use of covalent linkers can drastically alter the photosensitive properties of PS, thus decreasing its phototoxicity.^1^ Therefore, numerous studies on the use of nanoparticles, including dendrimers, as RB carriers have focused on noncovalent interactions,^5^ demonstrating the efficient intracellular uptake and superior photodynamic properties of such formulations.^16−18^ Because complex formation is usually based on ionic interactions, the process itself, as well as the physicochemical and biological properties of dendrimer/drug complexes, is greatly influenced by pH; ionic strength; buffer composition; and, most importantly, the structure of the dendritic carriers.^19,20^

In this study, we focused on well-characterized and commercially available cationic poly(amidoamine) (PAMAM) and poly(propyleneimine) (PPI) dendrimers of the third (G3) and fourth (G4) generation. We took a holistic approach, performing an in-depth characterization of dendrimer:RB interactions both *in vitro* and *in silico*, and performed further assessment of the multilevel biophysical and biological activity of the resultant complexes: singlet oxygen generation, cellular uptake, intracellular ROS production, and phototoxicity. To the best of our knowledge, this is the first attempt to compare the ability of cationic dendrimers of different types and generations to serve as carriers for anionic RB, and to link the dendrimer structure to the activity of complexes.

## 2 Results

### 2.1 *In Vitro* Evaluation of Dendrimer:RB Complexation

**Figure 1:**
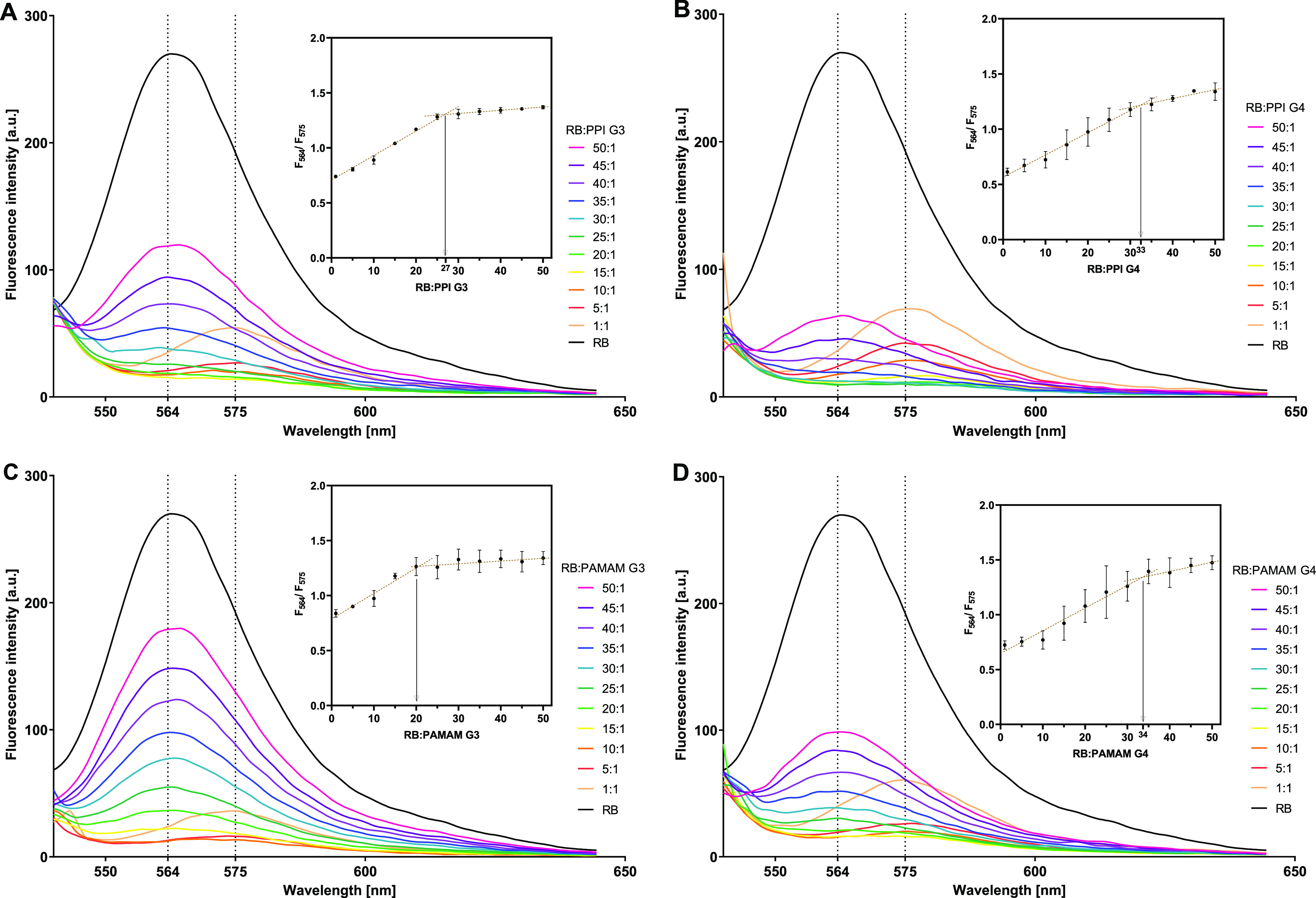
Changes in the fluorescence spectrum of RB (1 μM) upon titration with (A) PPI G3, (B) PPI G4, (C) PAMAM G3, and (D) PAMAM G4, maintaining a dendrimer:RB molar ratio of 1:50 to 1:1. The insets show the determination of the stoichiometry of complexes fully saturated with RB using Job’s method based on the plots of *F*_564_/*F*_575_ vs RB:dendrimer molar ratio. Data are presented as means ± SD; *n* = 3.

**Figure 2:**
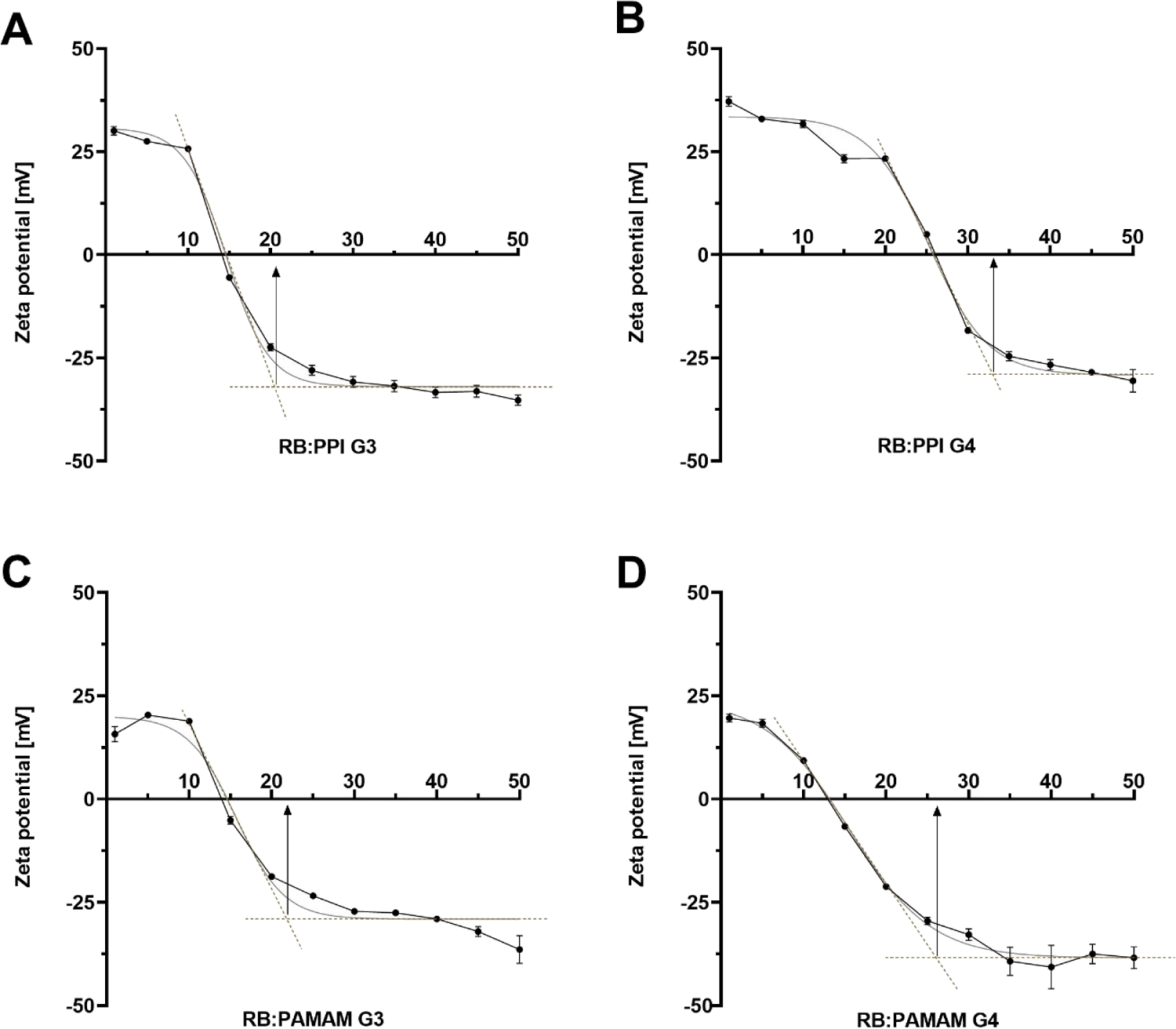
Titration curves for the measurements of zeta potential: effects of titration of 10 μM solutions of (A) PPI G3, (B) PPI G4, (C) PAMAM G3, and (D) PAMAM G4 with RB, maintaining the dendrimer:RB molar ratio of 1:1 to 1:50. Analysis of the course of titration curves allowed us to use Job’s method to determine the stoichiometry of complexes fully saturated with RB. Data are presented as means ± SD; *n* = 3.

To characterize the complex formation between the tested dendrimers and RB, we exploited their characteristic properties, i.e., dye fluorescence and the zeta potential of nanoparticles in solutions. Spectrofluorimetric studies revealed that the addition of a dendrimer to an RB solution caused a sharp reduction in dye fluorescence. Subsequent titration caused a progressive quenching of RB fluorescence until a red shift of the emission wavelength from 564 to 575 nm was observed, with a subsequent increase in the fluorescence signal (Figure 1), indicating polarity changes in the vicinity of the chromophore molecule.^21^ Based on this phenomenon, the *F*_564_/*F*_575_ ratio was calculated and plotted vs the RB:dendrimer molar ratio. Using Job’s method,^22^ we approximated the stoichiometry of binding in fully saturated complexes as 1:27 for PPI G3:RB, 1:33 for PPI G4:RB, 1:20 for PAMAM G3:RB, and 1:34 for PAMAM G4:RB (Figure 1, insets). This outcome was confirmed by the measurement of changes in the zeta potential of dendrimers during titration with RB. Upon the addition of subsequent portions of RB to the solution, the initial positive zeta potential of the dendrimers began to decrease until it reached a plateau at approximately −30 mV, indicating the full saturation of the polymers with PS. Based on the titration curves, we determined the stoichiometry of the formed complexes; the resultant values were similar to those obtained by spectrofluorimetric analyses: 1:21 for PPI G3:RB, 1:33 for PPI G4:RB, 1:22 for PAMAM G3:RB, and 1:26 for PAMAM G4:RB (Figure 2).

For the following experiments, the 1:10 dendrimer:RB molar ratio was used to ensure the stability of the complex and to maintain its positive surface potential, as positively charged nanoparticles have a greater ability to cross the barrier of biological membranes.^23,24^

### 2.2 *In Vitro* Photodynamic and Phototoxic Properties of RB and Dendrimer:RB Complexes

**Figure 3:**
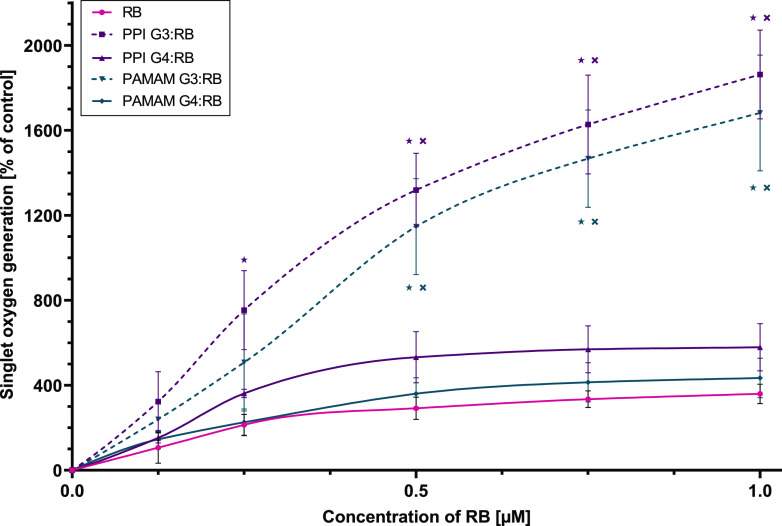
Singlet oxygen generation by RB and dendrimer:RB complexes in a 1:10 molar ratio. The singlet oxygen generation assay was performed using the ABDA probe as an indicator. Data are presented as the percentage of the singlet oxygen generation in the control sample containing only the ABDA probe; means ± SD; *n* = 4. *Statistically significant difference vs free RB (*p* < 0.05). ^×^Statistically significant difference between generations of dendrimers of the same type (*p* < 0.05).

Singlet oxygen generation assays using the ABDA probe showed that the tested compounds were able to increase the singlet oxygen levels. At the highest concentration tested, free RB caused a ∼3-fold increase in singlet oxygen generation relative to the control, slightly less than for the case of complexes with dendrimers of the fourth generation (∼4-fold for PAMAM G4 and ∼6-fold for PPI G4). On the other hand, complexes of RB with dendrimers of the third generation caused a greater increase in the generation of singlet oxygen (∼16-fold for PAMAM G3 and ∼19-fold for PPI G3), significantly exceeding the effect observed with free PS (Figure 3). Free dendrimers did not generate singlet oxygen (data not shown).

**Figure 4:**
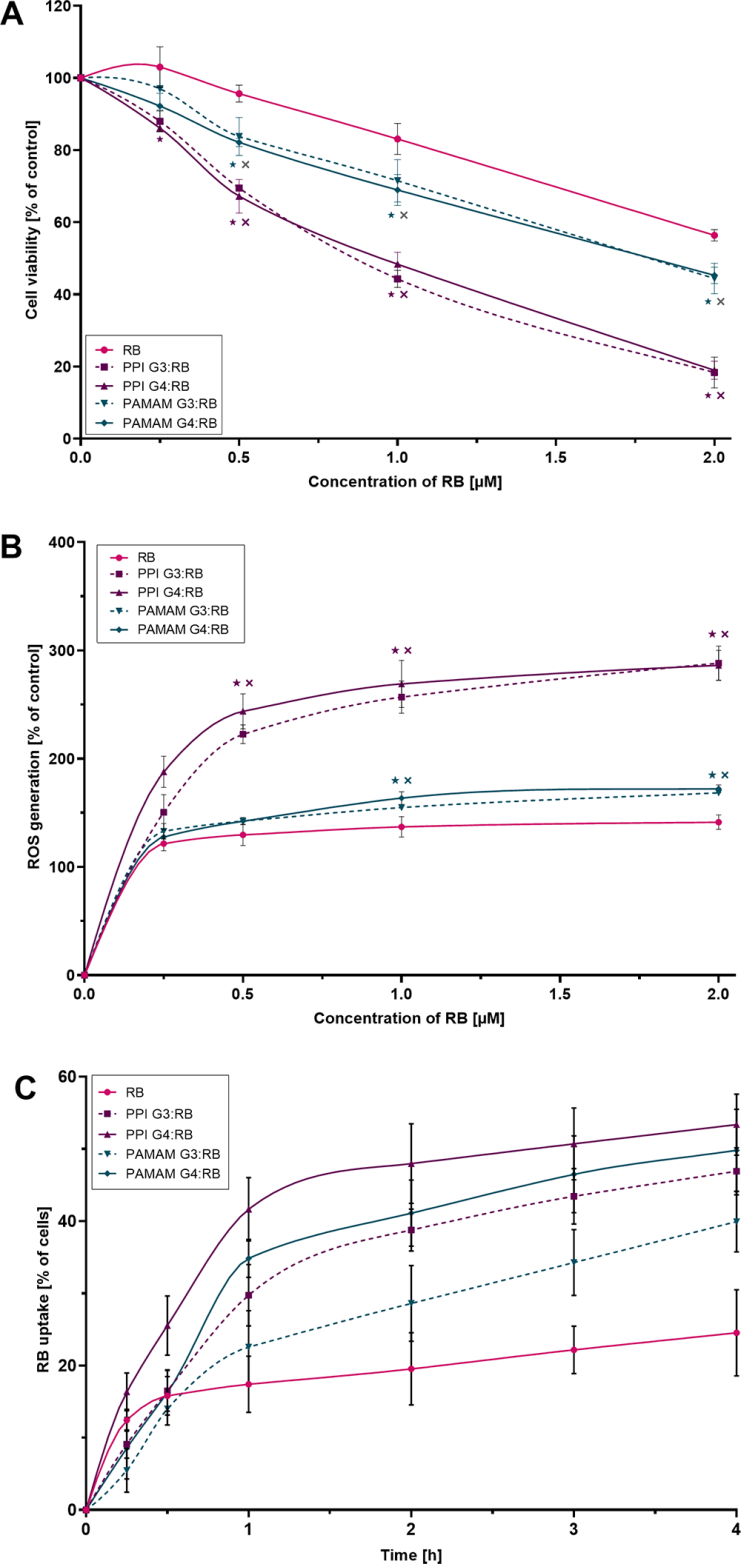
(A) Phototoxic effect of RB and dendrimer:RB complexes in 1:10 molar ratio in AsZ cells. Cell viability was determined using MTT assay. Data are presented as percentages of the viability of control (untreated) cells; means ± SD; *n* = 6. *Statistically significant difference vs free RB; *p* < 0.05. ^×^Statistically significant difference vs dendrimers of different type, regardless of generation; *p* < 0.05. (B) ROS production in AsZ cells triggered by RB and dendrimer:RB complexes in 1:10 molar ratio upon irradiation determined with the use of the 2′,7′-dichlorodihydrofluorescein diacetate (H_2_DCFDA) probe. Data are presented as percentages of intracellular ROS generation in control (untreated) cells; means ± SD; *n* = 4. *Statistically significant difference vs free RB; *p* < 0.05. ^×^Statistically significant difference vs dendrimers of different type, regardless of generation; *p* < 0.05. (C) Uptake of RB and dendrimer:RB complexes in 1:10 molar ratio by AsZ cells as determined by flow cytometry assay. Data are presented as the percentage of cells in the population exhibiting RB-associated fluorescence; means ± SD; n = 5. For statistical analysis, see Table S1.

The cytotoxicity of tested compounds was evaluated in three basal cell carcinoma cell line models, as basal cell carcinoma is the most common form of skin cancer and the most frequently occurring form of cancer overall.^25,26^ The complexes revealed a higher phototoxicity relative to free RB (Figure 4A and Figure S1), and this trend was maintained in all tested cell lines: RB in complex with PPI dendrimers was more toxic than RB in complex with PAMAM dendrimers, regardless of the generation. Cells treated with the free RB solution exhibited the highest viability. We did not observe cytotoxicity of free dendrimers or dark toxicity of RB and dendrimer:RB complexes (data not shown). The cell lines exhibited a range of susceptibilities to all treatments, with AsZ cells being the most susceptible [e.g., PPI:RB complexes with the highest RB concentration reduced the viability of AsZ cells to ∼20%; in the case of BsZ cells, viability was ∼30%, and for CsZ cells, viability was ∼50% (Figure 4A and Figure S1)]. Accordingly, we used AsZ for intracellular ROS production and cellular uptake assays.

The outcome of the intracellular ROS production assay coincided with the results of the cytotoxicity evaluation (Figure 4B). The tested compounds induced the production of ROS, with PPI:RB complexes exerting the greatest effect. The activity of the PAMAM:RB complexes was significantly lower but still exceeded the effect observed for free PS. The phenomenon was independent of the generation of dendrimers. Free dendrimers did not generate ROS (data not shown).

Complexation of RB with the tested dendrimers significantly increased the intracellular concentration of PS (Figure 4C). The PPI G4 dendrimer turned out to be the most effective carrier, with PAMAM G3 being the least efficient, but even in the latter case, the uptake of RB was almost 2-fold higher than when AsZ cells were treated with free PS. The effects of the PPI G3 and PAMAM G4 dendrimers were similar and intermediate between the PPI G4 and PAMAM G3. Overall, when comparing dendrimers of the same type, fourth-generation dendrimers had a greater ability to transport RB intracellularly than third-generation dendrimers. When comparing dendrimers of the same generation, PPI dendrimers were more efficient carriers than PAMAM dendrimers.

### 2.3 Molecular Modeling

#### 2.3.1 Single-Dendrimer Conformational Dynamics

We assessed the geometrical properties of dendrimers over the last 50 ns of two independent 200 ns MD simulations. The RoG, which represents a reliable metric for assessing the overall size of a dendrimer, and shape descriptors aspect ratio and asphericity (δ) were calculated as described in the Experimental Section. Geometrical properties of the two MD replicas were averaged over the last 50 ns of simulation, with snapshots taken every 2 ps (Table 1). The data obtained were in close agreement with *in silico* and experimental data from the previous literature for all the simulated systems (as reported in detail in Table S2), confirming that the dendrimer structures were well equilibrated.

**Table 1:** Radius of Gyration (RoG), Aspect Ratios, and Asphericity Values for the Simulated Dendrimers, Presented as Means ± SD

	RoG [nm]	*I_x_*/*I_y_*	*I_x_*/*I_z_*	δ
PAMAM G3	1.460 ± 0.058	0.708 ± 0.128	0.581 ± 0.087	0.026 ± 0.012
PAMAM G4	1.859 ± 0.064	0.839 ± 0.086	0.705 ± 0.069	0.012 ± 0.006
PPI G3	1.284 ± 0.024	0.792 ± 0.080	0.685 ± 0.068	0.013 ± 0.006
PPI G4	1.590 ± 0.020	0.826 ± 0.050	0.746 ± 0.042	0.008 ± 0.003

**Figure 5:**
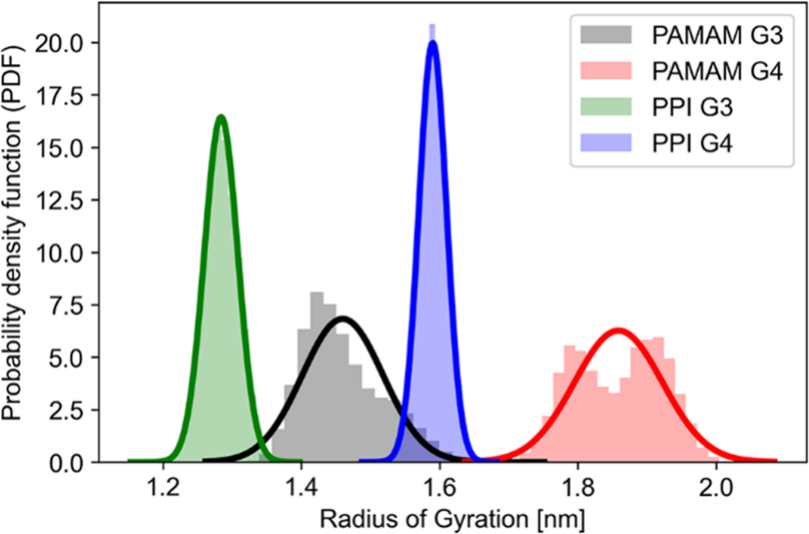
Probability density function (PDF) of the radius of gyration during the last 50 ns of two independent MD simulations.

Figure 5 shows the probability density function (PDF) of the RoG during the last 50 ns of MD replicas, highlighting the greater flexibility of PAMAM dendrimers relative to PPI dendrimers. Time series of the RoG during the entire simulations are reported in Figure S2.

#### 2.3.2 Dendrimer:RB Complexation and Interaction Dynamics

To assess the structural effects of RB on each dendrimer type and analyze the mode of dendrimer/drug interaction, dendrimer structures from the previous equilibration were simulated in the presence of 10 RB molecules (maintaining a 1:10 dendrimer:RB stoichiometry). MD trajectories showed the early and stable complexation of all 10 RB molecules after ≤16 ns, with no unbinding events observed throughout the 200 ns simulations (see also Figures S3–S5 and Videos S1–S4).

We assessed the structural effects of RB on the dendrimers again using RoG, aspect ratios, and asphericity measures, but we observed no remarkable effects upon ligand complexation (see Figure S6 and Figure S7). Similarly, the particle density of dendrimers with respect to the dendrimer central core was not remarkably altered in the presence of RB molecules (see Figure S8).

**Figure 6:**
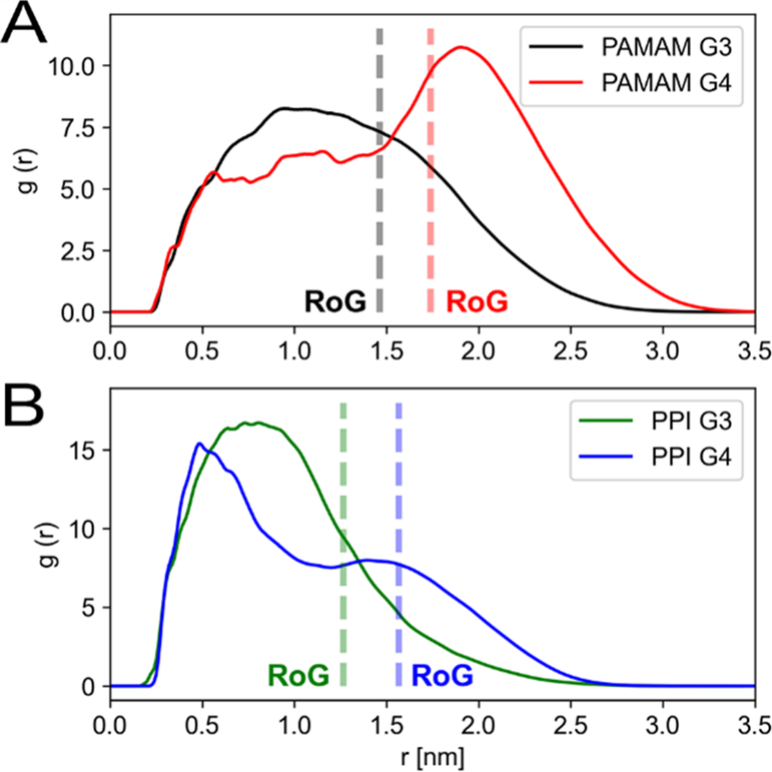
Radial distribution function (RDF) of RB with respect to the dendrimer central core for (A) PAMAM and (B) PPI dendrimers; dotted lines represent the radius of gyration for each dendrimer.

The radial distribution function (RDF) of the RB with respect to the dendrimer core (Figure 6) revealed that PPI dendrimers had a greater ability to internalize RB molecules. On the other hand, drug molecules were more exposed to the external solvent when bound to PAMAM dendrimers. It is worth mentioning that, despite this difference in ligand internalization, we observed no marked differences in the dendrimer:RB interaction surface among the dendrimers examined (see Figure S9).

**Figure 7:**
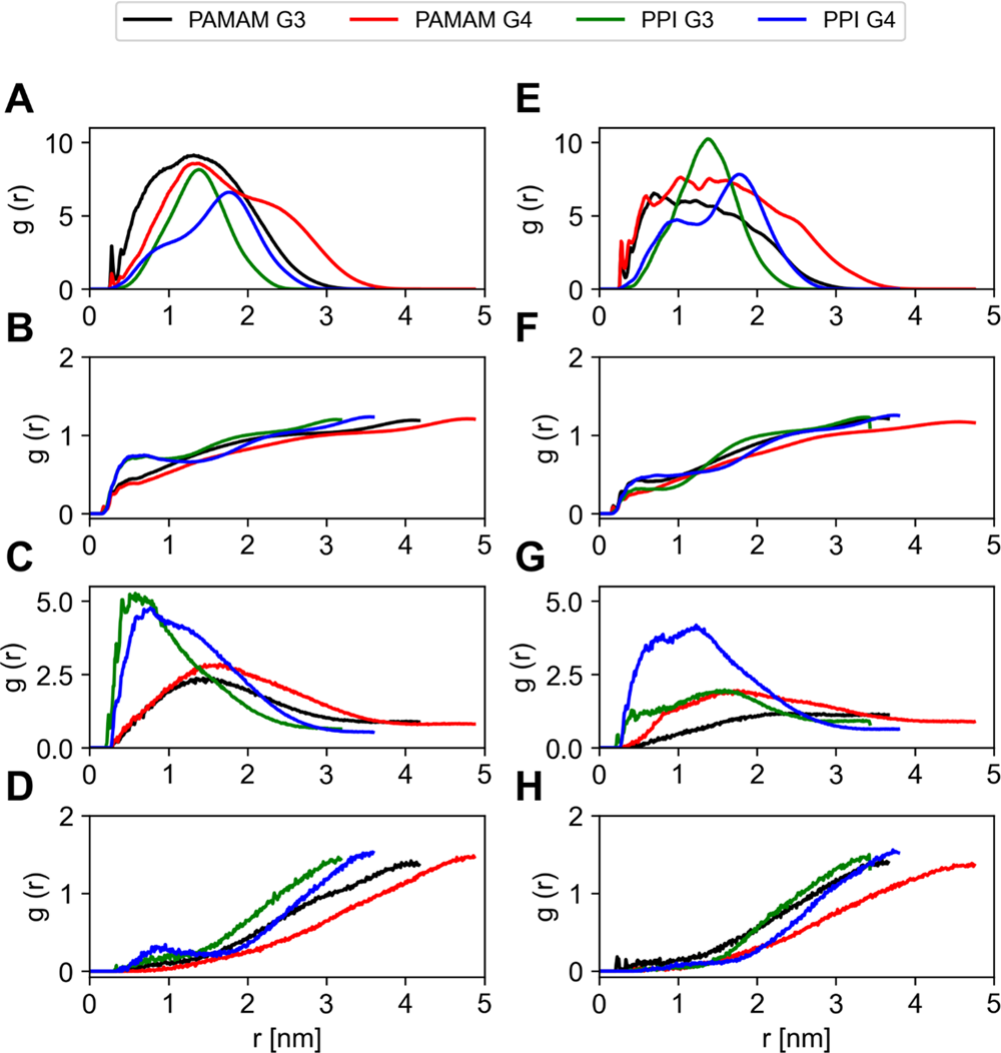
Radial distribution functions of external amines (A, E), TIP3P water (B, F), chlorine ions (C, G), and sodium ions (D, H) with respect to the dendrimer core from the concatenated trajectory of the last 50 ns of simulation of two independent MD replicas in the absence (A–D) and presence (E–H) of RB.

The RDFs for the external amino groups, water molecules, and chlorine and sodium ions are shown in Figure 7, in the presence and absence of RB, to compare the effects of the drug inclusion. The RDF trends of the external amines were unaltered in the presence of the RB for PPI dendrimers, confirming the more rigid behavior of these dendrimers (green and blue lines in Figure 7A,E). On the other hand, the RDF peaks of external amines of PAMAM dendrimers changed markedly upon drug complexation (black and red lines in Figure 7A,E), suggesting a major conformational change in the dendrimer structure. The reduced values of water molecules RDF in the internal layers are also indicative of the fact that these molecules are forced out by the entrance of RB, especially in the case of PPI dendrimers (Figure 7B,F). Similarly, the presence of RB leads to the ejection of chlorine ions from the internal layers of the dendrimers of the third generation (Figure 7C,G). The positively charged sodium ions on the other hand were not noticeably displaced with respect to the dendrimer core in the presence of RB if compared to the neat systems (Figure 7D,H).

**Figure 8:**
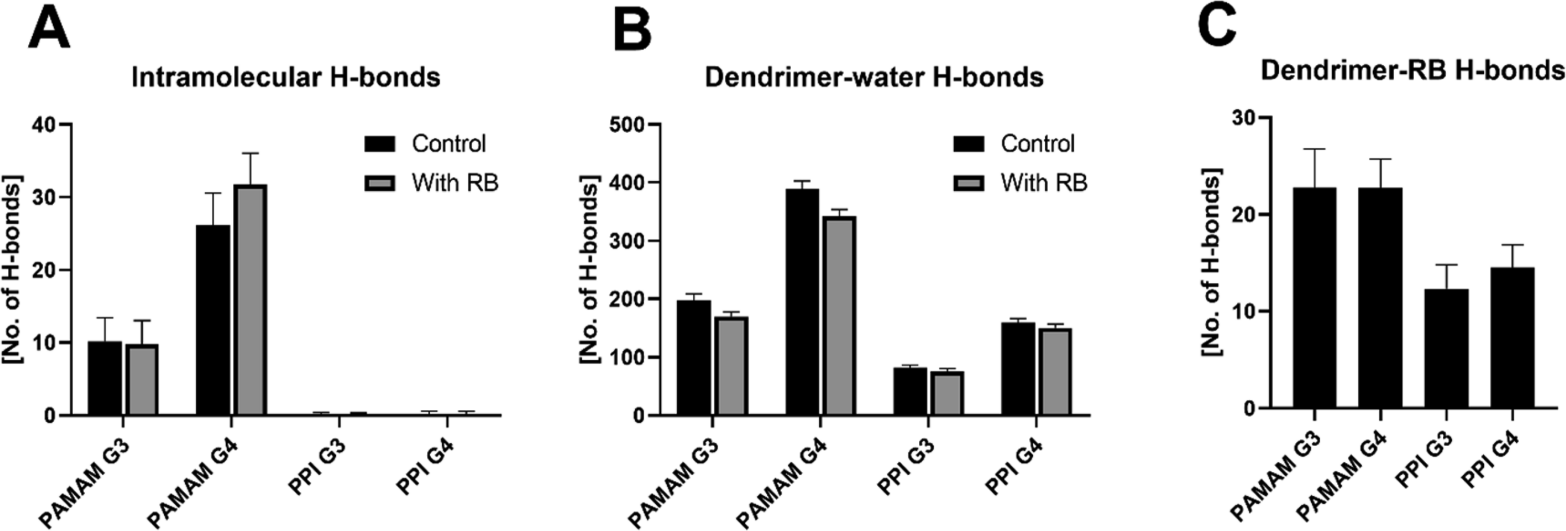
(A) Number of internal H-bonds in each dendrimer investigated. (B) Number of H-bonds between dendrimers and surrounding water molecules. (C) Number of H-bonds between dendrimers and RB. Data are presented as means ± SD across the last 50 ns of two 200 ns replicas.

We further assessed the structural characteristics of both the free dendrimers and their complexes with RB molecules by analyzing hydrogen bonds (H-bonds). As highlighted in Figure 8A, PAMAM dendrimers of both G3 and G4 are able to form an intramolecular network of H-bonds, mainly due to the presence of acceptor oxygen atoms within their underlying chemical structure.^27^ No intramolecular network of hydrogen bonds was observed for PPI dendrimers. Interestingly, the number of intramolecular H-bonds in PAMAM dendrimers did not seem to be influenced by the presence of RB. H-bonding between dendrimers and the surrounding water was more prominent in PAMAM dendrimers than in PPI dendrimers, with only a marginal decrease caused by RB complexation (Figure 8B).

Overall, PPI dendrimers formed fewer H-bonds with the solvent than PAMAM dendrimers, whereas fourth-generation dendrimers formed more H-bonds with the solvent, as expected from the increase in the number of surface amino groups. Finally, PAMAM dendrimers formed significantly more H-bonds with RB than PPI dendrimers, with no difference between dendrimer generations (Figure 8C). Overall, PAMAM dendrimers formed the largest number of H-bonds internally, with both the solvent and RB molecules.

**Figure 9:**
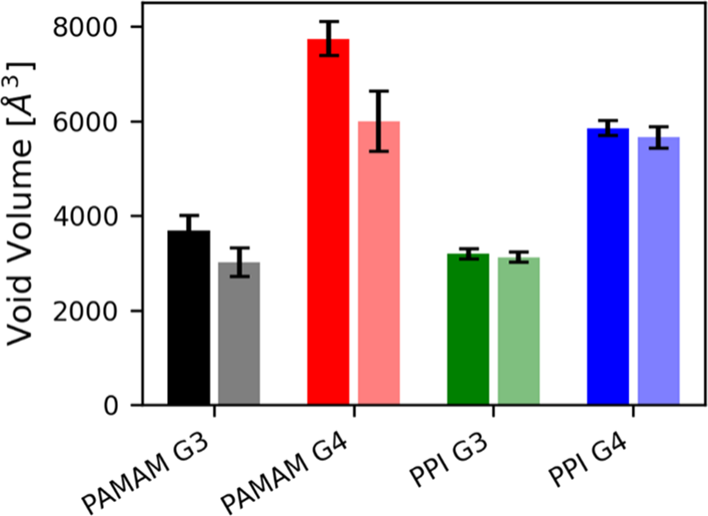
Volumes of dendrimers’ internal cavities. Solid colors refer to simulations of the free dendrimer systems, whereas shaded colors refer to simulations of the dendrimer:RB complexes.

Void volume analysis revealed that the presence of RB reduces the internal volumes of PAMAM dendrimers, whereas internal cavities of PPI dendrimers were not altered by the drug (Figure 9; see also Figure S10). Specifically, the ratios between the void volumes in the presence and absence of RB were 0.82 for PAMAM G3, 0.77 for PAMAM G4, 0.98 for PPI G3, and 0.97 for PPI G4.

**Figure 10:**
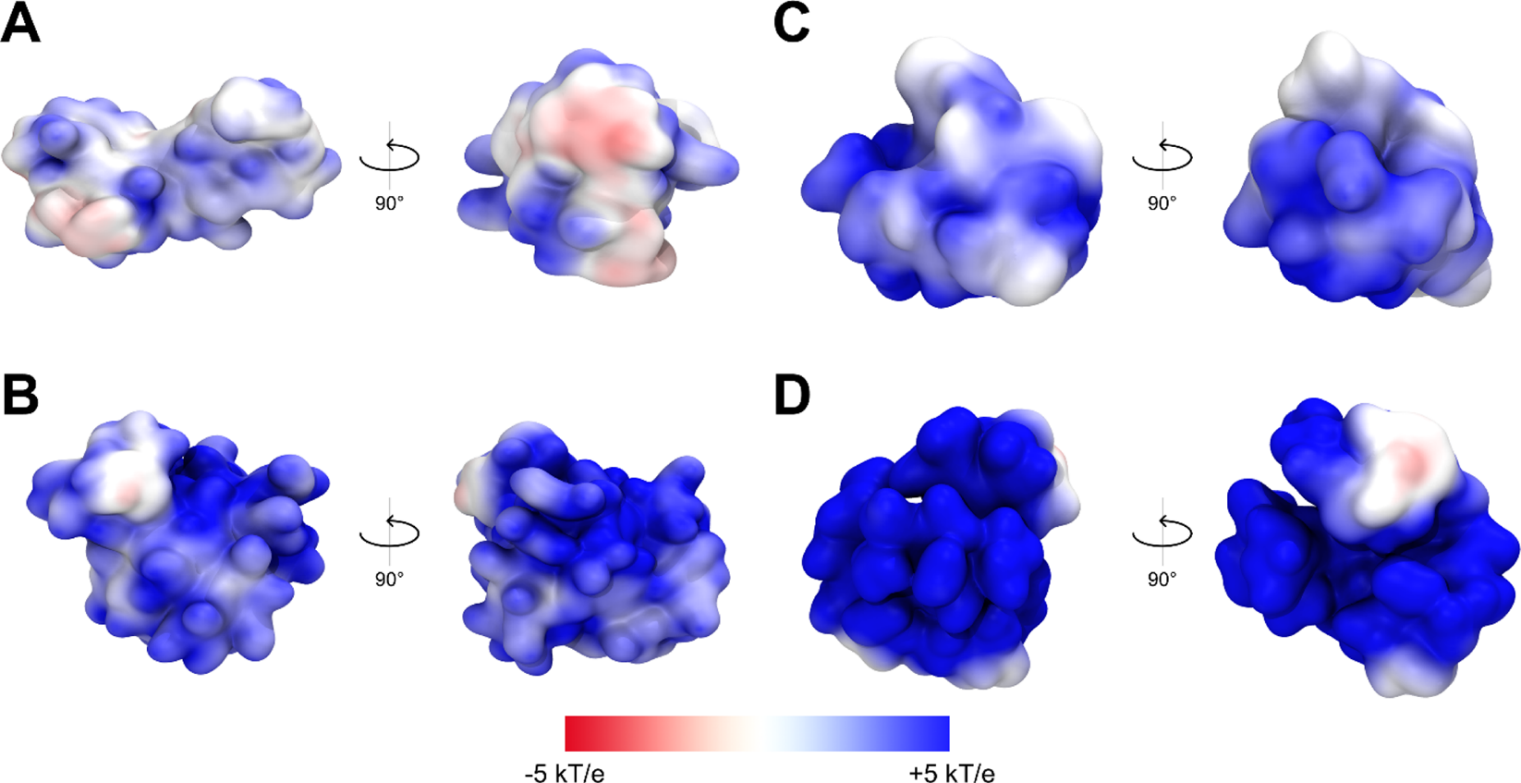
Front and side electrostatic maps for dendrimer:RB complexes (1:10): (A) PAMAM G3, (B) PAMAM G4, (C) PPI G3, and (D) PPI G4. Potential isocontours (obtained by the solution of the NLPBE at 150 mM ionic strength with a solute dielectric of 4 and solvent dielectric of 78.4) in the range from +5 kT/e (blue) to −5 kT/e (red).

Finally, we investigated the surface electrostatic potential of the complexes by extracting frames from the dendrimer:RB simulations and evaluating the dendrimer electrostatic potential in the presence of RB (Figure 10). We observed predominantly positive potential up to 5 kT/e on the dendrimer surface for all simulated systems; only PAMAM G3 had a prominent number of neutral surface patches (Figure 10A), indicating the ability of RB to locally neutralize the surface electrostatic potential of this specific dendrimer more effectively than for other systems. Overall, dendrimers of the fourth generation (Figure 10B,D) were characterized by a more positive surface potential even in the presence of bound RB, whereas third-generation dendrimers (Figure 10A,C) had a more neutral surface potential resulting from the shielding effect of bound RB.

## 3 Discussion and Conclusions

Photodynamic therapy (PDT), which relies on the use of a PS and a light source to induce singlet oxygen and ROS formation in the presence of molecular oxygen, is a promising therapeutic strategy against basal cell carcinoma. The use of dendrimers as drug carriers has the potential to overcome the known drawbacks of currently investigated PSs, such as self-quenching, short half-life, and suboptimal cellular uptake. In this work, we performed an in-depth characterization of the complexes of cationic poly(amidoamine) (PAMAM) and poly(propyleneimine) (PPI) dendrimers of the third and fourth generation with anionic rose bengal. A combined *in vitro* and *in silico* approach allowed for a complementary characterization of the effects of the dendrimers’ physical and chemical properties on their interactions with RB and ultimately on the phototoxic activity of the latter. Interestingly, most previous research concentrated on RB as a model molecule, which, due to its spectral properties, was used to study interactions with dendrimers (usually PAMAM; less often PPI and other types of macromolecules).^18,28−33^ Significantly fewer studies have analyzed the phototoxic activity of the dendrimer:RB complexes.^16−18^

The PAMAM and PPI dendrimers used in this study were inspected at atomic resolution at the single-dendrimer level. The analysis of neat dendrimer trajectories yielded geometrical shape descriptors consistent with the existing literature^34−50^ in terms of RoG, asphericity, and aspect ratios, implying well-converged simulations. Both types of dendrimers are spherical in shape. In general, G3 dendrimers have a smaller radius and smaller internal cavities than G4 macromolecules. When comparing dendrimers of the same generation, PPIs are smaller, more rigid, and more compact than PAMAMs. PAMAM dendrimers form intramolecular H-bonds (more in the case of the fourth generation), whereas PPI dendrimers do not; moreover, PAMAM dendrimers form more hydrogen bonds with water than PPI dendrimers.

In our initial studies of the formation of dendrimer:RB complexes and the determination of their stoichiometry, we analyzed the changes in the spectral properties of the dye upon complexation. As a result of the titration of the RB solution with dendrimers, the fluorescence intensity of RB decreased followed by a red shift of the spectral peak and subsequent increase in fluorescence. A similar red shift of both RB absorbance^31^ and fluorescence^32^ most often indicates the binding of the dye to the dendrimer surface.^21^ We exploited this phenomenon to determine binding stoichiometry. As we expected, G4 dendrimers could bind more RB molecules (approx. 35 per dendrimer molecule) than G3 dendrimers (20–25 RB molecules per dendrimer molecule), likely due to differences in the dendrimers’ volume and the level of protonation.^38,51,52^ The interactions of RB with the cationic phosphorus dendrimer were analyzed in an analogous manner, but the binding stoichiometry was significantly lower. This is probably due to the use of a different buffer (HEPES vs PBS) since it has been shown that the buffer composition has a significant influence on the formation of the complex^32,51^ Furthermore, stoichiometry was affected by NaCl concentration; consistent with our hypothesis, this indicates the essential role of electrostatic interactions in the formation of complexes between anionic RB and cationic dendrimers.^32^ These results were confirmed by Fourier transform infrared spectroscopy (FTIR). Additionally, RB does not form complexes with anionic phosphorus dendrimers.^33^ Other research groups also identified electrostatic interactions as the main driving force for the formation of complexes between RB and surface-modified PAMAM and PPI dendrimers^18,31^ and also reported a strong influence of the type of solvent on the binding stoichiometry.^18^

The determined stoichiometry of the PAMAM:RB and PPI:RB complexes was confirmed by titration of the dendrimer solutions with RB with the accompanying measurement of the zeta potential. The findings roughly coincided with those of the spectrofluorimetric method, with minor variations attributable to differences in the specificities of the two techniques. The results indicated that complexes fully saturated with RB exhibit negative zeta potential values. Assuming a surface binding mechanism, we can conclude that, in the final stages of titration, anionic RB molecules completely covered the outer layer of positively charged dendrimers.^20^ In light of these observations, in subsequent investigations, we set a subsaturating concentration of RB (namely, 1:10 dendrimer:RB molar ratio), which retained a residual positive surface charge for increased cellular uptake and decreased aggregation of complexes.^24^

Our molecular investigation of 1:10 dendrimer:RB complexes was carried out *in silico*, allowing the characterization of the binding mechanism and its effect on dendrimer geometry. Previous computational investigations of PPI:RB complexes clearly demonstrated the potential of atomistic simulations to complement experimental analyses by elucidating dendrimer:RB interaction dynamics.^53^ Herein, we extended the computational approach to substantially longer timescales, as well as to different dendrimer types and generations, and expanded the analysis by including a higher number of ligands as well as by randomizing their initial placement in the solvent. Although these differences hinder a direct comparison of the present and earlier results, the strong complexation of RB with positively charged dendrimers is confirmed. Indeed, MD simulations revealed short complexation times, below 16 ns, and the ability of the investigated dendrimers to carry all 10 RB molecules, with no subsequent unbinding event detected over 200 ns in each MD replica, suggesting binding energies significantly exceeding thermal fluctuation (kT) and a strong tendency of RB to bind to each type of dendrimer. This behavior was primarily driven by electrostatics (see also Figure S11), consistent with previous observations. Interestingly, despite the predominant role of electrostatic interactions, we also observed the formation of H-bonds between dendrimers and RB, more strongly in the case of PAMAM than PPI.

The binding of RB did not significantly affect the geometrical characteristics of the dendrimers, and the estimated dendrimer:RB interaction areas were similar in all investigated systems. The volumes of the internal cavities decreased in the case of PAMAM dendrimers while remaining unchanged for PPI dendrimers. This was also reflected in the arrangement of the surface amino groups, which was influenced by RB binding only for PAMAM dendrimers. The attachment of RB also caused the displacement of water molecules (more evident in case of PPI dendrimers) and negatively charged chlorine ions (in the case of G3 dendrimers) from the inside of the dendritic scaffolds.

Notably, we found that RB has the ability to penetrate the structure of dendrimers, positioning itself preferentially inside the scaffold rather than on the surface. Comparison of these findings with the previously discussed fluorescence red shift indicates that the dendrimer:RB binding mechanism is more complex than indicated solely by spectrofluorimetric studies. Overall, the *in silico* investigation highlighted the greater ability of PPI dendrimers to internalize RB molecules within the inner dendrimer branches (see RDF data, Figure 6B). The size of the dendrimers and the specific arrangement of the RB molecules also influenced the surface potential of the complexes, which was significantly reduced (to values close to neutral) in the case of G3 dendrimers. Given the characteristics of the surface potential, it is plausible that interactions among multiple dendrimers occur in the presence of RB. This idea is consistent with preliminary data concerning interacting systems consisting of two dendrimers and 20 RB molecules, in which G3 complexes exhibited a marked tendency to engage in dendrimer–dendrimer interactions (see Figure S12 and Videos S5 and S6). Interestingly, the complexes with PPI G4 also showed a tendency to aggregate during longer measurement times, which was consistent with the results of the analysis of the hydrodynamic diameter of the complexes by dynamic light scattering (DLS) (Table S3).

Our approach allowed us to highlight significant differences in complex formation and interaction patterns as a function of dendrimer type and generation. Because the photodynamic properties of RB are determined by several factors, including the chemical environment, it seems reasonable that these observed differences could significantly influence the ultimate cytotoxic effect.

Because the level of singlet oxygen generation is thought to be directly related to the efficacy of photodynamic therapy,^54^ we assessed the activity of tested compounds in this regard. RB complexes with G3 dendrimers exhibited a significantly higher production of singlet oxygen, whereas the effect of G4 dendrimers was only slightly higher than that of free RB. At the same time, free dendrimers did not generate singlet oxygen. A similar effect was previously observed for supramolecular complexes of PSs and various polymers,^55^ including RB and cationic dendrimers.^16^ On the other hand, no increase in singlet oxygen production was observed in the case of RB complexed with anionic half-generation PAMAM dendrimers;^17^ for PEG2000-modified PPI and PAMAM G4 dendrimers, the singlet oxygen level was reduced upon encapsulation of RB. In the latter case, however, the effect was attributed to RB aggregation and quenching due to the high local concentration of PS inside dendrimers (approx. 180 RB molecules per dendrimer).^18^ Such complexes exhibited no increase in phototoxic activity (relative to free RB) in HeLa cells. These observations underlie the influence of both the dendrimer:RB interaction and their molar ratio on the ultimate photodynamic effect.

The increase in singlet oxygen production can be explained by the immobilization of RB by the nanoparticle in more than one dimension, translating into a change in optical properties. Analysis of the Jablonski diagram reveals that excited RB can return to the ground state through photon emission or the transition to the triplet excited state responsible for singlet oxygen generation.^1^ Considering the decrease in fluorescence during RB binding by the tested dendrimers, it is likely that, in this case, the second process is favored.^56^ Nanoparticles can affect the fluorescence of the dye in the solution in several ways, including the internal fluorescence filter effect, dynamic quenching, static quenching, surface enhancement, and modulation of the quantum yield of the fluorophore. These phenomena are related to the binding-induced conformational changes in the structure of PS.^57,58^ Furthermore, the patterns of interaction between the dye, nanoparticle, and solvent can significantly affect aggregation, causing changes in the behavior and properties of PS in the vicinity of different nanoparticles suspended in the same solvent.

Indeed, the effect of dendrimer binding on RB-triggered singlet oxygen generation might be directly linked to the fact that RB tends to aggregate under physiological conditions^59^ due to π-stacking and that PS aggregation has a detrimental effect on singlet oxygen generation due to the self-quenching of excited states.^60^ Hence, better encapsulation of individual RB molecules by dendrimers would lead to a reduction in RB–RB aggregation and thus of self-quenching, yielding more efficient generation of singlet oxygen.

From this standpoint, the difference in the generation of singlet oxygen by G3 and G4 complexes is worth noting and remains difficult to explain at this stage of our research. The difference may be associated with the better prevention of RB aggregation and improved stabilization of the excited state by G3 dendrimers. Further stabilization of the transition state might also be achieved through complex–complex interactions. In addition, given the observed displacement of anions from inside the G3 dendrimers caused by RB, we can assume that anions play an active thermodynamic role in RB binding; thus, the latter might be more favored for G3 than G4 dendrimers. Such a binding strength- and mode-dependent production of singlet oxygen by PSs has already been observed during interactions with DNA.^61^ These observations highlight the need for further biophysical analyses, including confirmation using more direct, probe-independent methods of singlet oxygen detection; such techniques are currently under development in our laboratory.

Surprisingly, the results of the singlet oxygen generation assay were not reflected in our studies of cellular models. In these analyses, we observed the highest phototoxic activity in basal cell carcinoma models using PPI:RB complexes, regardless of generation. A similar lack of dependence on generation was observed in the case of PAMAM:RB complexes, whose cytotoxicity was intermediate between the action of PPI:RB complexes and free RB. It should be emphasized that, in the tested concentration range, free dendrimers did not exhibit phototoxicity, and no dark toxicity was observed for any of the compounds examined. Similar results were obtained when analyzing the production of intracellular ROS. Because the cellular factor is the most important difference between the singlet oxygen generation assay and subsequent studies, we hypothesized that the differences observed in cellular models are related to another crucial aspect of RB application: cellular uptake and subcellular localization. Indeed, dendrimer:RB complexes were able to deliver PS intracellularly much more effectively than intracellular transport of free RB.^16^

The efficiency of the intracellular transport of complexes perfectly matched the differences in their surface potential, evaluated based on the APBS electrostatic map analysis (Figure 10) of dendrimer:RB MD simulations: the PAMAM G3:RB complex with the surface electrostatic potential closest to neutral was the least efficient carrier, whereas the most cationic PPI G4:RB complex had the greatest intracellular transport capacity. These observations are consistent with reports of the efficient crossing of cell membranes by positively charged nanoparticles and allow us to predict the behavior of complexes depending on their surface potential.^23,24,62,63^

The different delivery capacities of the investigated dendrimers may also be related to their chemical composition, as well as mechanical and structural properties. In this regard, PAMAM and PPI dendrimers exhibited differences in flexibility throughout the MD simulations, with PPIs exhibiting higher rigidity than PAMAMs. This behavior was emphasized by (i) the RDF, which showed that external amines of PPI dendrimers were not affected by the presence of RB; (ii) the RoG, which indicated that PAMAM dendrimers were more flexible; and (iii) the void volume, which revealed that the volumes of PPI internal cavities were not affected by the inclusion of the drug. Thus, the rigid and compact structure of PPI dendrimers may favor the intracellular delivery of RB.

On its own, the more efficient singlet oxygen generation is insufficient to explain the ultimate effects on cell viability, as the efficacy of PDT also depends on the cellular uptake and subcellular location of PS.^64^ Indeed, because the generation of singlet oxygen outside the cell is unlikely to significantly affect cell viability due to the limited lifespan of singlet oxygen molecules,^64^ the ability of dendrimers to efficiently cross the cell membrane might be a decisive factor. Therefore, the observed cytotoxic effect, likely related to the production of intracellular ROS, may be the result of an increase in the cellular RB uptake and production of singlet oxygen. The latter effect, in turn, may differ significantly between cellular and extracellular systems due to the difference in light penetration and the changes in properties of the complexes upon transfer from a buffer with a limited composition into the culture medium and subsequently into the cell interior.

The joint effects of dendrimer structural and mechanical properties, the tendency of RB to penetrate the dendrimer, and the dendrimer surface electrostatics are thus crucial factors determining the ability of complexes to induce cell death. Based on our results, we conclude that cationic PAMAM and PPI dendrimers can serve as efficient carriers of RB in photodynamic therapy. Due to their structural properties, the patterns of interaction with RB, and the characteristic features of the dendrimer:RB complexes, PPI dendrimers outperform PAMAM dendrimers, providing the most efficient uptake in the case of PPI G4 and significantly increasing generation of singlet oxygen in the case of PPI G3. Particular attention should be paid to the selection of appropriate drug and dendrimer concentrations, ensuring a uniform distribution of RB within the structure of the dendrimer, thus preventing the aggregation of the PS and allowing the maintenance of a positive surface charge of the delivery system.

## 4 Experimental Section

### 4.1 Materials

The RB, fetal bovine serum (FBS), penicillin/streptomycin solution, trypsin–EDTA solution, ABDA probe [9,10-antherachenediyl-bis(methylene) dimalonic acid], MTT [3-(4,5-dimethyl-2-thiazolyl)-2,5-diphenyl-2H-tetrazolium bromide], and HEPES (4-(2-hydroxyethyl)-1-piperazineethanesulfonic acid) were purchased from Sigma-Aldrich (Taufkirchen, Germany). Dulbecco’s phosphate-buffered saline without calcium and magnesium (DPBS) was purchased from Biowest (Nuaillé, France). HBSS (Hanks’ balanced salt solution) and the 154 CF culture medium were obtained from Gibco/ThermoFisher Scientific (Waltham, MA, USA). Chelex 100 Resin was obtained from Bio-Rad (Hercules, CA, USA). H_2_DCFDA (2′,7′-dichlorodihydrofluorescein diacetate) was purchased from Invitrogen/ThermoFisher Scientific. Dimethyl sulfoxide (DMSO) was purchased from POCH (Gliwice, Poland). Murine basal cell carcinoma lines (AsZ, BsZ, and CsZ) were provided by Dr. Ervin Epstein (Children’s Oakland Research Institute, Oakland, CA, USA).

Poly(propyleneimine) (PPI) dendrimers of the third and fourth generation^a^ with 32 or 64 primary amino surface groups, respectively, were obtained from Symo-Chem (Eindhoven, the Netherlands). Poly(amidoamine) (PAMAM) dendrimers of the third and fourth generation with 32 and 64 primary amino surface groups, respectively, were obtained from Sigma-Aldrich.

### 4.2 Methods

#### 4.2.1 Spectrofluorimetric and Zeta Potential Studies on the Interaction between PAMAM or PPI Dendrimers and RB

Fluorescence (F) emission spectra were obtained on an LS 55 fluorescence spectrometer (PerkinElmer, Waltham, MA, USA) at a constant temperature of 25 °C. All samples were prepared in HEPES buffer (10 mM, pH 7.4) and measured in quartz cuvettes. The excitation wavelength was set to 525 nm, and spectra were recorded between 540 and 650 nm. Excitation and emission slits were 5 and 7.5 nm, respectively. The RB solution in a constant concentration of 1 μM was titrated with dendrimer solutions in concentrations ranging from 0.02 to 1 μM to maintain the molar ratio of dendrimer:RB complexes between 1:50 and 1:1. The experiments were performed in three independent replicates. To determine the stoichiometry of the polymer/dye complexes, plots of *F*_564_/*F*_575_ vs the RB:dendrimer molar ratio were evaluated using Job’s method.

Zeta potential measurements were performed using electrophoretic mobility assays on a Zetasizer Nano ZS (Malvern Instruments Ltd., Malvern, UK) at a constant temperature of 25 °C. All samples were prepared in a HEPES buffer (10 mM, pH 7.4). Dendrimer solutions of constant concentration (10 μM) were placed in DTS 1070 folded capillary cells, and their zeta potentials were measured. The solutions were subsequently titrated with RB solution to obtain final RB concentrations ranging from 10 to 500 μM, corresponding to dendrimer:RB molar ratios of 1:1 to 1:50. The experiments were performed in three independent replicates. Analysis of the titration curves for all studied systems enabled the evaluation of the stoichiometry of complexes as follows: the decreasing dependence of dendrimer zeta potential on dendrimer:RB mixture stoichiometry was extrapolated to the intersection with the eventual zeta potential value of the fully saturated dendrimer, and binding stoichiometry was determined from the intersection point (Job’s method).

#### 4.2.2 Preparation of Complexes for Further *In Vitro* Studies

Dendrimers were dissolved in double-distilled water to a final concentration of 40 μM. Dendrimer solutions were prepared fresh and used on the same day. RB (dissolved in double-distilled water) was added to the dendrimer solutions in a dendrimer:RB molar ratio of 1:10 (to a final RB concentration of 400 μM). This molar ratio ensures the complete complexation of RB molecules by all tested dendrimers. The mixtures were stirred for 0.5 h at the ambient temperature. Stock solutions were prepared just before the experiments.

#### 4.2.3 Singlet Oxygen Generation Assay

The singlet oxygen generation was studied using the ABDA probe (final concentration: 5 μM) as an indicator. Solutions of RB, PAMAM G3:RB, PAMAM G4:RB, PPI G3:RB, PPI G4:RB, and free dendrimers in the highest concentration used for complex formation (0.1 μM) were prepared in 10 mM HEPES. The complexes were prepared at RB concentrations of 0.125, 0.25, 0.5, 0.75, and 1 μM. Upon sample preparation, 100 μL of each solution was transferred to a 96-well black plate. All measurements were performed on a fluorescence microplate reader (Fluoroskan Ascent FL, ThermoFisher Scientific) at an excitation wavelength of 355 nm and an emission wavelength of 430 nm. Samples were mixed before each measurement. The first measurement was recorded without the ABDA probe to determine whether RB, dendrimers, or their complexes exhibit any fluorescence in this range. Following the first measurement, ABDA was added to each well, and the fluorescence of the probe without irradiation was measured. Next, the plate was immediately placed under a Q.Light Pro Unit lamp (Q.Light, Rorschach, Switzerland) equipped with a filter emitting visible light in the wavelength range 385–780 nm. Fluorescence was measured in 5 min intervals during irradiation for 5–60 min. The experiments were performed in four independent replicates. The slopes of the fluorescence curves were considered to be a measurement of singlet oxygen generation. The results were presented as percentages of the singlet oxygen generation in the control sample (HEPES buffer irradiated with probe).

#### 4.2.4 Cell Culture

AsZ, BsZ, and CsZ (murine basal cell carcinoma) cell lines were cultured in the 154 CF medium with 5% penicillin/streptomycin, 0.05 mM calcium, and 2% Chelex-purified, heat-inactivated FBS. Cells were cultured in T-75 culture flasks at 37 °C/5% CO_2_ and subcultured every 2 or 3 days. Cells were harvested using 0.25% (w/v) trypsin/0.03% (w/v) EDTA. The number of viable cells was determined by Trypan blue exclusion assay on a Countess Automated Cell Counter (Invitrogen, Carlsbad, CA, USA).

#### 4.2.5 Cytotoxicity Studies

AsZ, BsZ, and CsZ cells were seeded in 96-well transparent plates at a density of 3 × 10^4^ cells per well in 90 μL of the medium and incubated for 24 h before experiments. Then, using stock solutions (according to 4.2.2), the samples (PAMAM G3:RB, PAMAM G4:RB, PPI G3:RB PPI G4:RB, and free RB solutions) were prepared in the HEPES buffer and added to the cells to obtain final RB concentrations of 0.25, 0.5, 0.75, and 1 μM. The cytotoxicity of free dendrimers was also evaluated at the highest concentration used for the preparation of complexes. Cells were incubated with tested compounds for 5 h (37 °C, 5% CO_2_). The medium was replaced with DPBS, and the cells were irradiated with visible light using the Q.Light Pro Unit lamp for 30 min. Immediately after irradiation, DPBS was replaced with the fresh culture medium, and the cells were incubated for 24 h (post-PDT incubation). The so-called ″dark″ toxicity (without irradiation) was evaluated in parallel.

The cell viability was measured by the MTT assay. MTT was added to the wells at a final concentration of 0.5 mg/mL, and the plates were incubated for 2 h (37 °C, 5% CO_2_). After incubation, formazan crystals were dissolved in DMSO, and the absorbance was read at 570 nm using a PowerWave HT Microplate Spectrophotometer (BioTek, Winooski, VT, USA). Experiments were performed in six independent replicates. Cell viabilities are presented as percentages of the viability in the untreated control.

#### 4.2.6 ROS Generation Assay

An H_2_DCFDA probe was used to investigate the intracellular production of ROS. For this purpose, AsZ cells were seeded in 96-well black plates at a density of 1 × 10^4^ cells per well in 90 μL of the medium. After incubation for 24 h, the samples (PAMAM G3:RB, PAMAM G4:RB, PPI G3:RB PPI G4:RB, and free RB solutions) were prepared in the HEPES buffer and added to the cells to obtain final RB concentrations of 0.25, 0.50, 1, or 2 μM. The ROS-generating activity of free dendrimers was also evaluated at the highest concentration used for the preparation of complexes. Cells were incubated with tested compounds for 5 h (37 °C, 5% CO_2_). The medium containing the tested compounds was removed, a 2 μM solution of H_2_DCFDA in HBSS was added to each well, and the plates were incubated for the next 20 min in the dark (37 °C, 5% CO_2_). Next, the cells were washed with HBSS, and the background fluorescence (excitation: 485 nm; emission: 530 nm) of nonirradiated cells submerged in 100 μL HBSS was measured on a PowerWave HT Microplate reader (BioTek). The cells were then irradiated using Q.Light Pro Unit lamp for 30 min, and 2′,7′-dichlorofluorescein (DCF) fluorescence was measured. The experiments were performed in four independent replicates. The ROS level was calculated as the DCF fluorescence intensity and was presented as a percentage of the ROS production in control samples (without treatment). Each measurement was corrected by subtraction of the background fluorescence intensity (before irradiation).

#### 4.2.7 Cellular Uptake Assay

AsZ cells were seeded into 24-well plates at a density of 1 × 10^5^ cells per well and incubated for 24 h (37 °C, 5% CO_2_). Next, RB, PAMAM G3:RB, PAMAM G4:RB, PPI G3:RB, and PPI G4:RB (5 μM final concentration of RB) were added to each well, and the cells were incubated with the compounds for up to 4 h. Following incubation, the compounds were removed, and the cells were washed with DPBS and detached using the trypsin–EDTA solution. The fresh culture medium was added to the cells, and the samples were gently mixed and collected for measurements. To estimate cellular uptake, the fluorescence of the samples was measured using flow cytometry (LSRII, Becton Dickinson, Franklin Lakes, NJ, USA). The excitation and emission filters were 520 and 570 nm, respectively. The experiments were performed in five independent replicates. The results are presented as the percentage of cells in the population that internalized RB.

#### 4.2.8 Statistical Analysis

Statistical significance was tested using two-way ANOVA for concentrations and compound series followed by *post hoc* Tukey’s test for pairwise difference testing. In all tests, *p* values <0.05 were considered statistically significant. Data were collected from at least three independent experiments and presented as arithmetic means ± SD.

#### 4.2.9 *In Silico* Studies

##### 4.2.9.1 System Setup

Initial configurations for PAMAM and PPI dendrimers were built using the Dendrimer Builder Toolkit (DBT)^34^ and the General Amber Force Field (GAFF).^66^ The protonation state was chosen based on neutral pH, as reported previously.^34,35,43,67^ Under these conditions, the amine groups in the external layers of PAMAM dendrimers were fully protonated, whereas all the primary amines present at the periphery and the tertiary amines in alternating layers of the PPI dendrimers were protonated, resulting in 2/3 protonation according to the Ising model.^52,68^ The assigned protonation states resulted in a total charge of +32, +64, +42, and +84 for PAMAM G3, PAMAM G4, PPI G3, and PPI G4, respectively. RB was described by the GAFF force field, and partial charges were assigned using the AM1-BCC charge method (see also Figure S13).^69^ Topology and parametrization were constructed using antechamber and GROMACS tools.^70,71^

##### 4.2.9.2 Single-Dendrimer Conformational Dynamics

Each dendrimer was positioned in a dodecahedral box filled with TIP3P (transferable intermolecular potential 3P) water molecules^72^ and ions to neutralize the system charge at a physiological NaCl concentration (0.15 M). Each system was energy-minimized using the steepest descent energy minimization algorithm (2000 steps). After randomly initializing atom velocities following a Maxwell–Boltzmann distribution, a 100 ps position-restrained molecular dynamics (MD) was performed in the canonical ensemble (NVT) at 300 K using the v-rescale algorithm^73^ for temperature coupling. Then, an NPT position-restrained MD was executed for 500 ps using a v-rescale thermostat^73^ and a Berendsen barostat^74^ to equilibrate temperature (300 K) and pressure (1 atm), respectively. Finally, an unrestrained 200 ns MD simulation was performed in the isothermal–isobaric ensemble (NPT) at 300 K and 1 atm using the v-rescale and Parrinello–Rahman coupling algorithms.^73,75^ The GROMACS 2020 package was used for all MD simulations.^76^ Long-range electrostatic interactions were calculated at every step with the particle mesh Ewald method^77^ with a cutoff radius of 1.2 nm; the same cutoff was also applied to Lennard–Jones interactions. The simulation time step was 2 fs using the LINCS (LINear Constraint Solver) algorithm.^78^ To ensure the reproducibility of the data, a second replicate was performed after re-initializing velocities after the minimization step and following the same simulation protocol as described above. The final 50 ns of MD simulations was considered as a single ensemble trajectory representing the structural stability of each treated system.

##### 4.2.9.3 Dendrimer:RB Complexation and Interaction Dynamics

The final configuration from the aforementioned equilibrium ensembles was extracted for each dendrimer type. The structure was again inserted into a dodecahedral box, and 10 RB molecules were added in random positions around the dendrimer to obtain a 1:10 molar concentration ratio. The box was filled with TIP3P water molecules and NaCl at a physiological concentration (0.15 M) to neutralize the system charge. The systems were then simulated using the same simulation protocol described in the previous section. Two replicates were performed to ensure data reproducibility, and the last 50 ns of these MD simulations was considered as a single ensemble trajectory representing the structural stability of each investigated system.

##### 4.2.9.4 Simulation Analysis

As reported previously,^34,35,43,48^ the geometrical characterization of the investigated dendrimers was evaluated using the radius of gyration (RoG), which measures the size of the dendrimers, and three main geometrical descriptors (*I_x_*/*I_y_*, *I_x_*/*I_z_*, and δ) that evaluate the shape of the dendrimers. In more detail, we calculated the three principal momenta of inertia (*I_x_*, *I_y_*, and *I_z_*) and derived two aspect ratios (*I_x_*/*I_y_* and *I_x_*/*I_z_*) and asphericity (δ) as defined by Rudnick and Gaspari:^79^
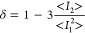
1where *I*_1_ = *I_x_* + *I_y_* + *I_z_*, *I*_2_ = *I_x_I_y_* + *I_y_I_z_* + *I_x_I_z_*, and angle brackets denote time averaging. In this formulation, the closer to zero the value of δ is, the more spherical the molecule is.

The volumes of dendrimer internal cavities were calculated as described previously.^80,81^ First, volumes associated with accessible surface areas (*V*_sasa_) were calculated at different probe radii. Then, a linear fitting on the cubic root values of *V*_sasa_ was performed at different probe radii, starting from 0.4 nm. The deviation of the calculated volume from the aforementioned fitting line, at a probe radius of 0.3 nm, provides an estimate of the volumes of internal voids. Internal cavities have been evaluated both for the neat dendrimer systems and for the dendrimer:RB complexes. In the latter case, to ensure a consistent comparison, the volumes of dendrimer cavities were evaluated after removing RB molecules from the complex snapshots, thus excluding the volume occupied by RB molecules from the calculations. This ensures that we evaluated the actual structural effects on the dendrimer itself rather than the volume occupancy of RB.

We also analyzed the dendrimer:RB complexes by comparing electrostatic potentials in the absence and presence of bound RB using the APBS package.^82^ Specifically, the nonlinear Poisson–Boltzmann equation was applied using single Debye–Huckel sphere boundary conditions on a 200 × 200 × 200 grid with a spacing of 1 Å centered at the center of mass (CoM) of the molecular system. The relative dielectric constants of the solute and the solvent were set to 4 and 78.4,^82,83^ respectively. The ionic strength was set to 150 mM, and the temperature was fixed at 300 K.

The visual inspection of simulations and all molecular renderings was carried out with the Visual Molecular Dynamics (VMD) package.^84^

## Supporting Information Available

Additional figures and tables illustrating the cytotoxicity, cellular uptake, and further results of *in silico* studies (PDF)
MD trajectories showing the early and stable complexation of all 10 RB molecules (MP4)
MD trajectories showing the early and stable complexation of all 10 RB molecules (MP4)
MD trajectories showing the early and stable complexation of all 10 RB molecules (MP4)
MD trajectories showing the early and stable complexation of all 10 RB molecules (MP4)
G3 complexes exhibiting a marked tendency to engage in dendrimer–dendrimer interactions (MP4)
G3 complexes exhibiting a marked tendency to engage in dendrimer–dendrimer interactions (MP4)
Molecular formula strings (CSV)
PDB submission (ZIP)

## Abbreviations

DCF: 2′,7′-dichlorofluorescein
DLS: dynamic light scattering
H_2_DCFDA: 2′,7′-dichlorodihydrofluorescein diacetate
LINCS: LINear Constraint Solver
MD: molecular dynamics
NPT: isothermal–isobaric ensemble
NVT: canonical ensemble
PAMAM G3:RB: complex of PAMAM dendrimer third generation with rose bengal
PAMAM G4:RB: complex of PAMAM dendrimer fourth generation with rose bengal
PDF: probability density function
PPI G3:RB: complex of PPI dendrimer third generation with rose bengal
PPI G4:RB: complex of PPI dendrimer fourth generation with rose bengal
PS: photosensitizer
RB: rose bengal
RDF: radial distribution function
RoG: radius of gyration
TIP3P: transferable intermolecular potential 3P

## Footnotes

a According to ref (65), the nomenclature for Tomalia-type PAMAM dendrimers can be used for PPI dendrimers. Hence, we adopted the suggested classification, describing the commercially available PPI dendrimer of the fifth generation (DAB-Am-64) as fourth generation, and fourth generation (DAB-Am-32) as third generation.
