# Supplementary material for: Noncovalent Interactions with PAMAM and PPI Dendrimers Promote the Cellular Uptake and Photodynamic Activity of Rose Bengal: The Role of the Dendrimer Structure
Source: J Med Chem. 2021 Sep 21;64(21):15758–71. doi: 10.1021/acs.jmedchem.1c01080 (PMC8591609; doi:10.1021/acs.jmedchem.1c01080)
Supplement: Supplementary file 1 — jm1c01080_si_001.pdf [file jm1c01080_si_001.pdf]

Supporting Information

Noncovalent interactions with PAMAM and PPI dendrimers promote cellular uptake and photodynamic activity of rose bengal: The role of the dendrimer structure

Krzysztof Sztandera[1], Michał Gorzkiewicz[1], Ana Sofia Dias Martins[2], Lorenzo Pallante[3], Eric Adriano Zizzi[3], Marcello Miceli[3], Mateusz Bątal[1], Catarina Pinto Reis[2,4], Marco A. Deriu[3], Barbara Klajnert-Maculewicz[1*]*

[1] Department of General Biophysics, Faculty of Biology and Environmental Protection, University of Lodz, 141/143 Pomorska St., 90-236 Lodz, Poland.

[2] iMed.ULisboa – Research Institute for Medicines, Faculdade de Farmácia, Universidade de Lisboa, Av. Prof. Gama Pinto, 1649-003 Lisboa, Portugal

[3] Polito^{BIO}MedLab, Department of Mechanical and Aerospace Engineering, Politecnico di Torino, Corso Duca degli Abruzzi 24, 10129 Turin, Italy

[4] Instituto de Biofísica e Engenharia Biomédica, Faculdade de Ciências, Universidade de Lisboa, Campo Grande, 1749-016 Lisboa, Portugal

*Cooresponding authors:

krzysztof.sztandera@edu.uni.lodz.pl, barbara.klajnert@biol.uni.lodz.pl

Table of contents

	Page
Figure S1	S3
Table S1	S3
Table S2	S4
Figure S2	S5
Figure S3	S5
Figure S4	S6
Figure S5	S7
Figure S6	S8
Figure S7	S8
Figure S8	S9
Video S1, S2, S3, S4	S9
Figure S9	S9
Figure S10	S10
Figure S11	S10
Multiple-dendrimer systems	S10
Figure S12	S11
Video S5, S6	S11
Hydrodynamic diameter measurement	S11
Table S3	S11
Figure S13	S12
References	S12

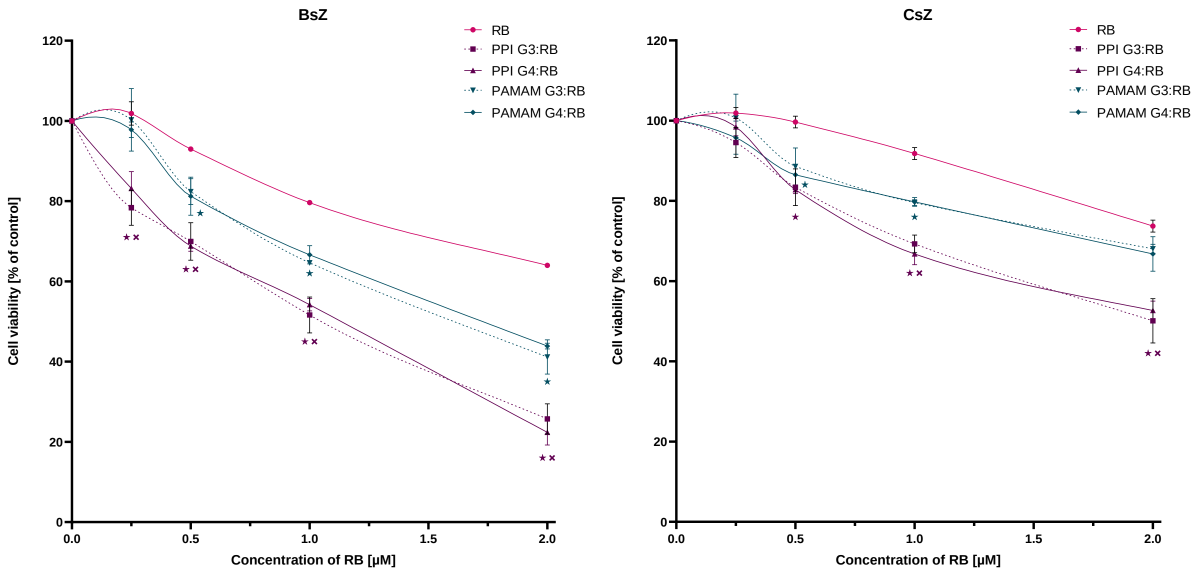

Figure S1. *Phototoxic effect of RB and dendrimer:RB complexes in 1:10 molar ratio, in BsZ and CsZ cells. The viability of cells was determined using MTT assay. Data are presented as percentage of viability of control (untreated) cells; mean ± SD; n = 6. *Statistically significant difference vs. free RB; $p < 0.05$. ×Statistically significant difference vs. dendrimers of different type, regardless of generation; $p < 0.05$.*

Table S1. *Statistically significant differences (*p < 0.05) in intracellular uptake of the tested compounds at the indicated time points.*

		RB	PPI G3:RB	PPI G4:RB	PAMAM G3:RB	PAMAM G4:RB
0.25 h	RB	-			*	
	PPI G3:RB		-	*		
	PPI G4:RB			-		
	PAMAM G3:RB				-	
	PAMAM G4:RB					-
0.5 h	RB	-		*		
	PPI G3:RB		-	*		
	PPI G4:RB			-	*	*
	PAMAM G3:RB				-	
	PAMAM G4:RB					-
1 h	RB	-	*	*		*
	PPI G3:RB		-	*	*	
	PPI G4:RB			-	*	*
	PAMAM G3:RB				-	*
	PAMAM G4:RB					-
2 h	RB	-	*	*	*	*
	PPI G3:RB		-	*	*	

	**PPI G4:RB**			-	*	*
	**PAMAM G3:RB**				-	*
	**PAMAM G4:RB**					-
**3 h**	**RB**	-	*	*	*	*
	**PPI G3:RB**		-	*	*	
	**PPI G4:RB**			-	*	
	**PAMAM G3:RB**				-	*
	**PAMAM G4:RB**					-
**4 h**	**RB**	-	*	*	*	*
	**PPI G3:RB**		-	*	*	
	**PPI G4:RB**			-	*	
	**PAMAM G3:RB**				-	*
	**PAMAM G4:RB**					-

Table S2*. Comparison of RoG values obtained in the present work with previously published computational and experimental data.*

		**Literature (MD)**			**Experimental**	
**System**	**This work**	**GAFF**	**CHARMM**	**Other**	**SANS**	**SAXS**
**PAMAM G3**	1.460 ± 0.058	1.578 ± 0.029[1]<br>1.21[2]<br>1.52[3]	1.533[4]<br>1.408[5]	1.61 ± 0.01[6]<br>1.454[7]<br>1.97[8]<br>1.619[5]	1.666[9]	1.509[10]
**PAMAM G4**	1.859 ± 0.064	2.064 ± 0.022[1]<br>1.49[2]	2.104[4]	2.06 ±0.01[6]<br>2.143 ± 0.01[11]<br>2.17 ± 0.001[8]<br>1.848[7]<br>1.7[12]<br>2.67[8]	2.129[9]	1.860[10]
**PPI G3**	1.284 ± 0.024			1.16 ± 0.007[6]<br>1.342[7]	1.24[13]<br>1.16[14]	1.33[15]
**PPI G4**	1.590 ± 0.020	1.601 ± 0.011[1]<br>1.577 ± 0.021[16]		1.40 ± 0.007[6]<br>1.648[7]<br>1.59[17]<br>1.577 ± 0.021[16]	1.56[13]<br>1.39[14]	1.43[15]

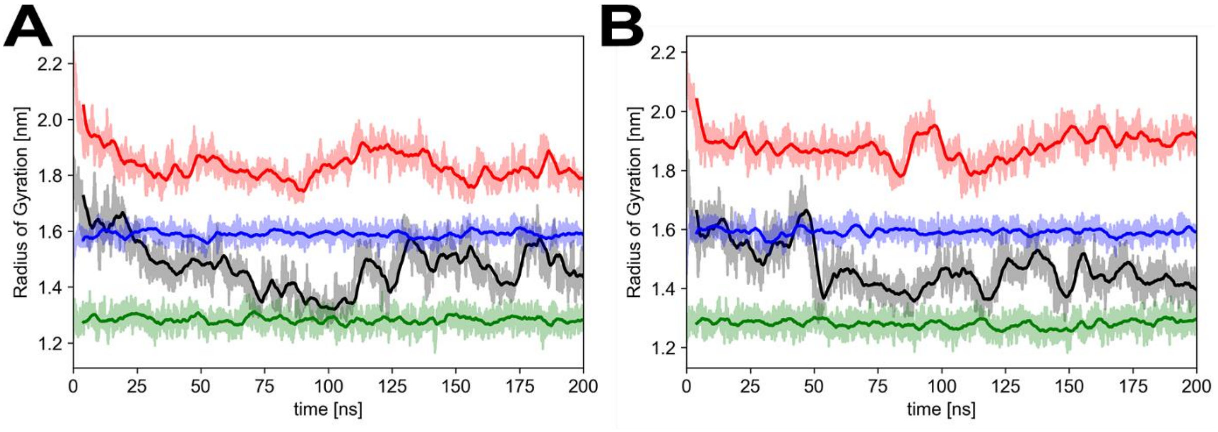

Figure S2. *Radius of gyration (RoG) during the first (A) and second (B) 200 ns replicates of the dendrimer systems. PAMAM G3 is presented in black, PAMAM G4 in red, PPI G3 in green, and PPI G4 in blue. Shaded colors show data from all processed trajectories, whereas solid colors represent moving averages.*

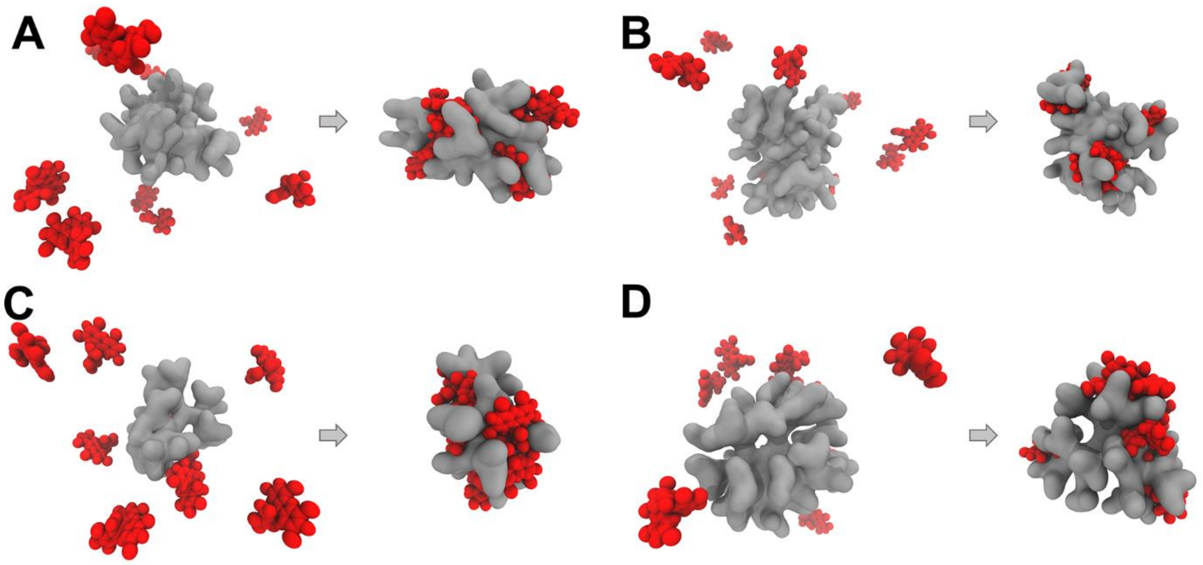

Figure S3. *Starting and final snapshots of the MD simulation from the first replica for (A) PAMAM G3, (B) PAMAM G4, (C) PPI G3 and (D) PPI G4 with 10 RB molecules.*

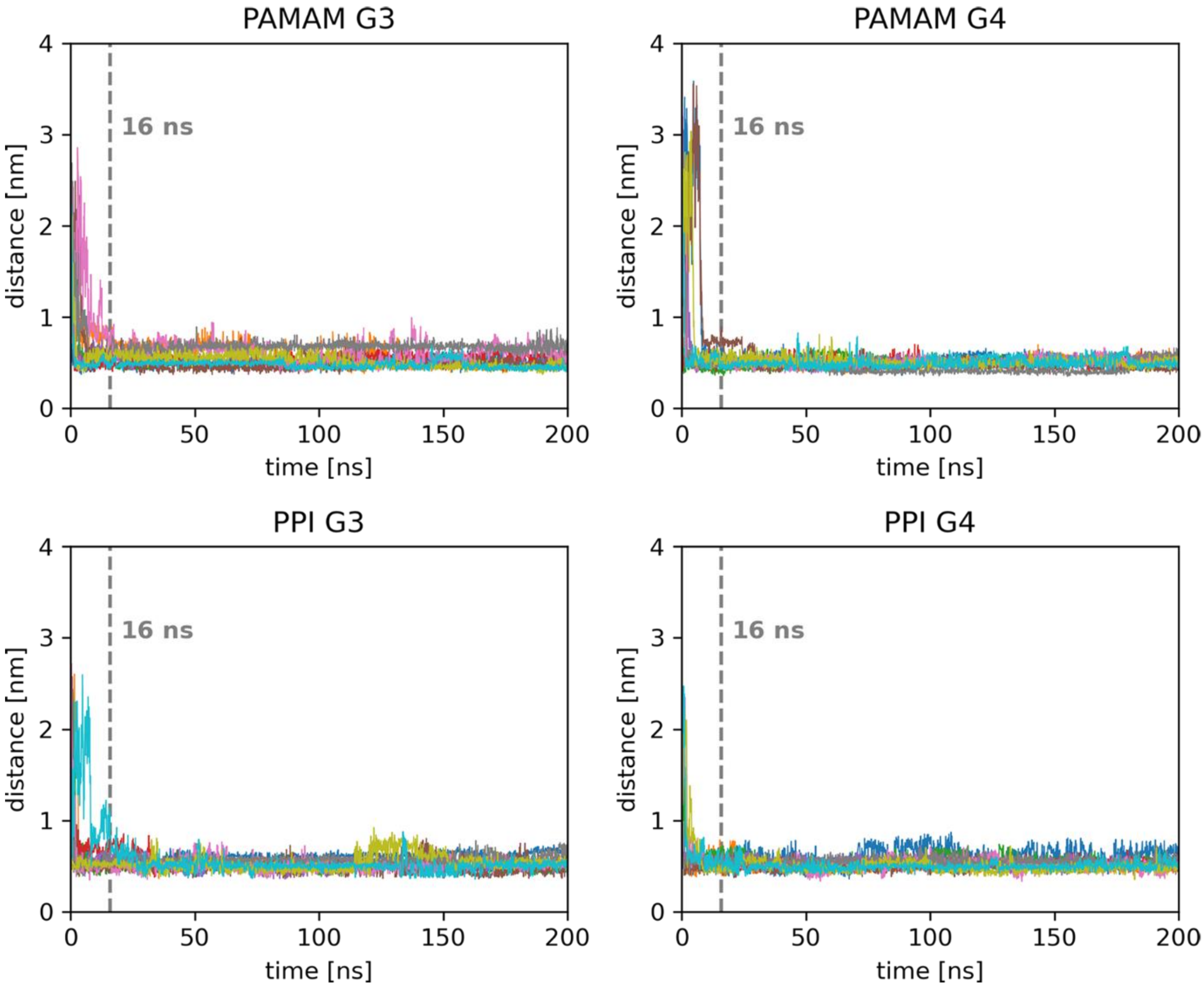

Figure S4. *Distances of the centers of mass of the 10 RB molecules with respect to the center of mass of each dendrimer during the first MD replica. Each RB molecule is represented with a different color.*

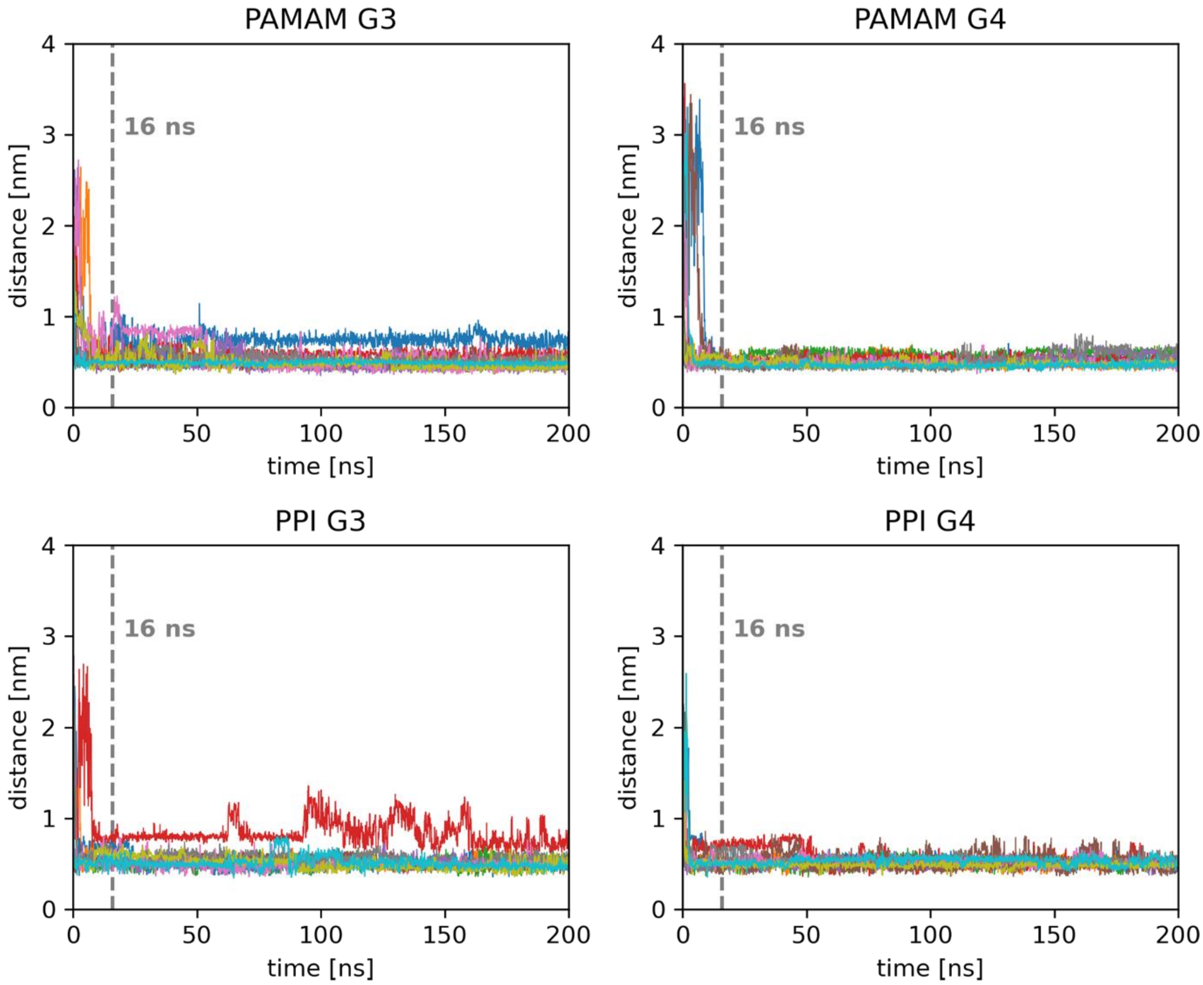

Figure S5. *Distances of the centers of mass of the 10 RB molecules with respect to the center of mass of each dendrimer during the second MD replica. Each RB molecule is represented with a different color.*

Video S1, S2, S3, S4. *Representative trajectories of the 200 ns MD simulations of RB binding to PAMAM G3, PAMAM G4, PPI G3, and PPI G4, respectively.*

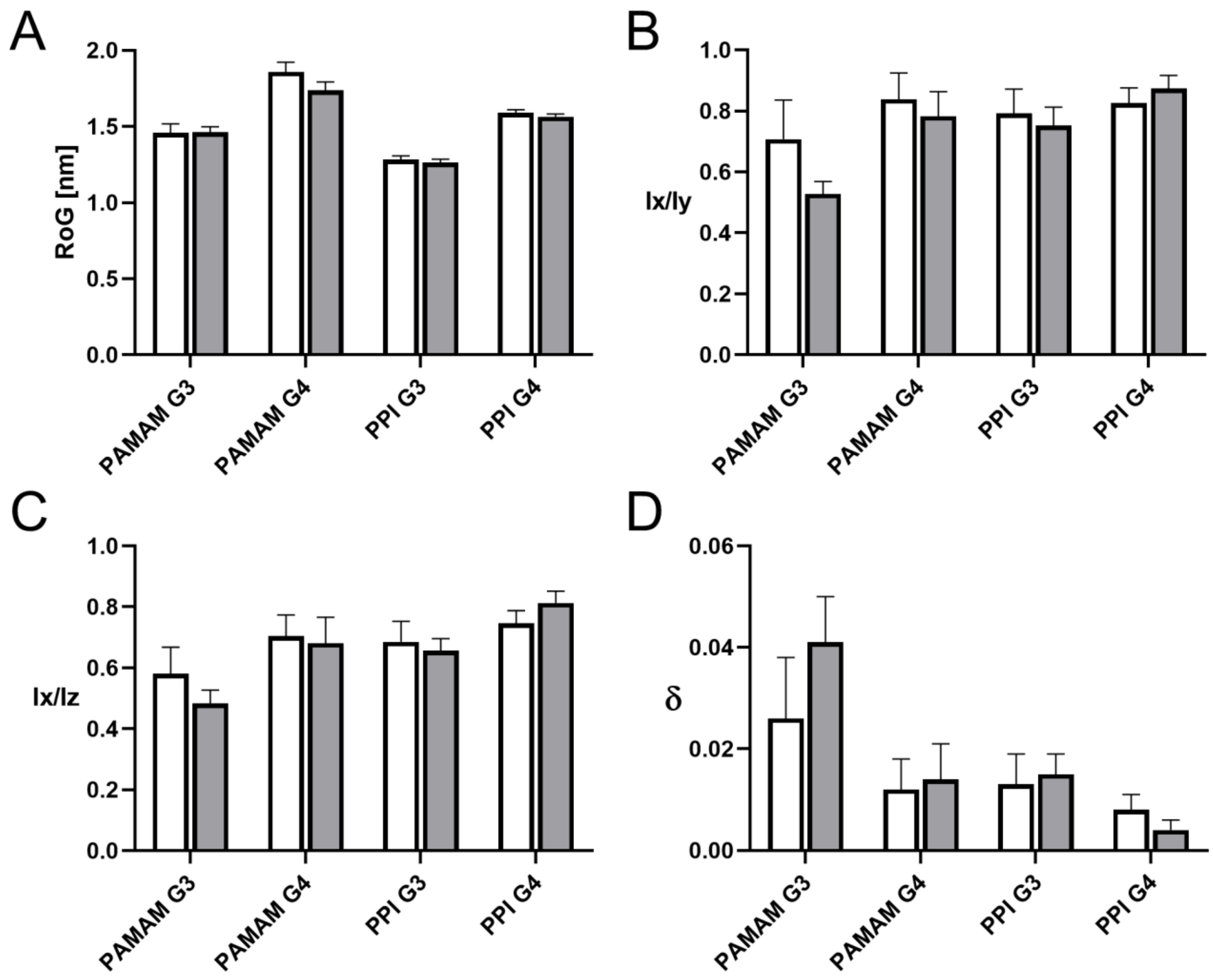

Figure S6. *Comparison of average values (± SD) of geometrical descriptors in the presence (gray) and absence (white) of RB. (A) Radius of gyration (RoG), (B, C) aspect ratios, and (D) asphericity. Data were obtained by averaging over the last 50 ns of simulation from two MD replicas, with snapshots taken every 2 ps.*

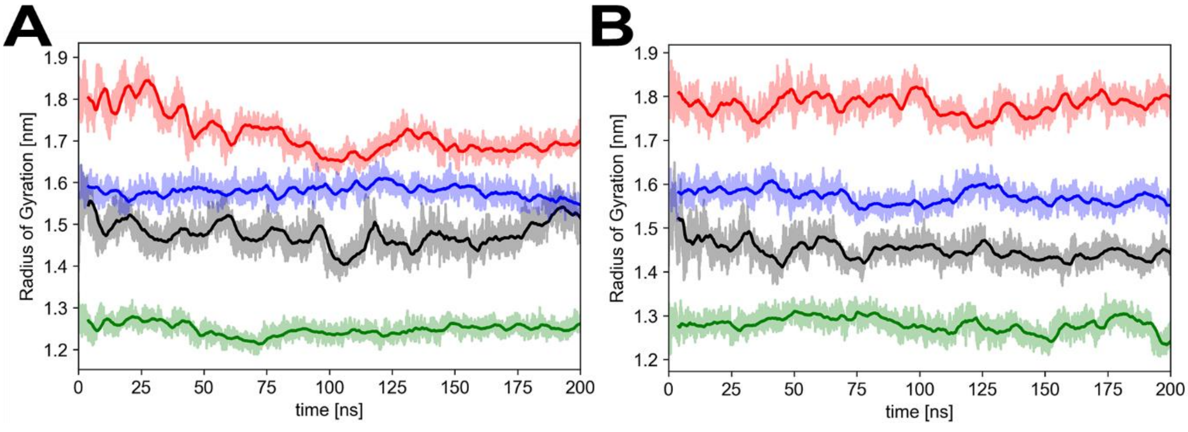

Figure S7. *Radius of gyration (RoG) during the first (A) and second (B) 200 ns replicates of the dendrimer:RB complexes. PAMAM G3:RB complex is presented in black, PAMAM G4:RB in red, PPI*

G3:RB in green, and PPI G4:RB in blue. Shaded colors show data from all processed trajectories, whereas solid colors represent moving averages.

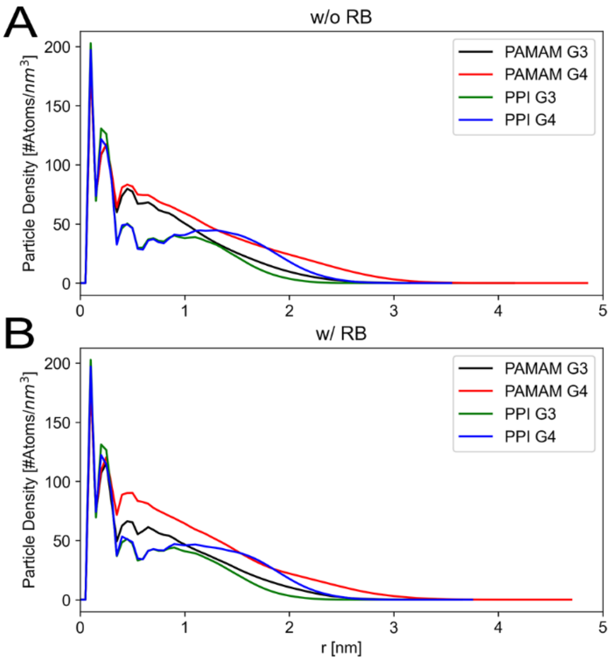

Figure S8. *Particle density (number of atoms per unit volume) of the investigated dendrimers with respect to the dendrimer central core in the (A) presence and (B) absence of RB.*

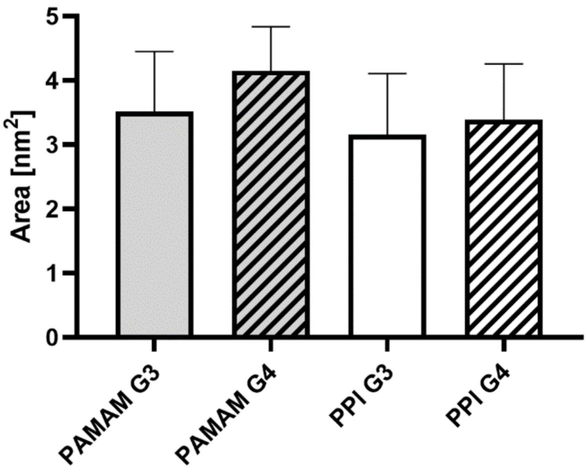

Figure S9. *Average (± SD) of the interaction area between dendrimers and RB molecules during the last 50 ns of the two independent MD replicates.*

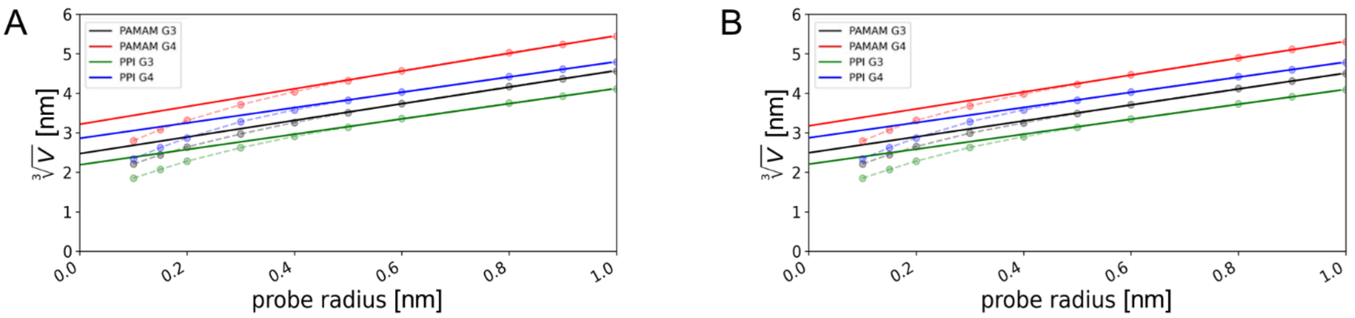

Figure S10. *Linear regression to calculate the volumes of internal cavities for (A) dendrimer-only systems and (B) dendrimer:RB complexes.*

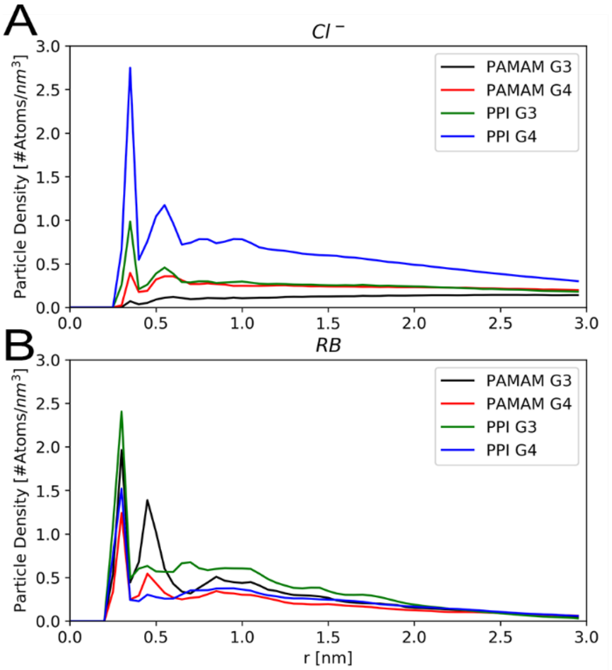

Figure S11. *RDF of (**A**) the CL^- ions and (**B**) RB molecules with respect to the positively charged amines of the simulated dendrimers during the concatenated equilibrium trajectory (last 50 ns of each MD replica).*

Multiple-dendrimer systems

Snapshots of each of the simulated dendrimers at structural equilibrium were extracted from previously described MD simulations and used to build multiple-dendrimer systems as follows: two copies of each dendrimer configuration were juxtaposed to obtain two-dendrimer systems with an inter-dendrimer distance of 2 nm. The obtained configuration was inserted into a dodecahedral box, and 20 RB molecules were added in random positions around the dendrimer to obtain a 1:10 dendrimer:RB molar ratio. The systems were solvated, neutralized, and simulated following the protocols described in the Materials and Methods.

During this set of simulations, G3 dendrimers exhibited a marked tendency to interact with each other, whereas G4 dendrimers either interacted on longer time scales or were unable to interact (**Figure S12** and **Video S5** and **S6**). We are well aware that the complete characterization of such processes requires a more thorough sampling. Accordingly, we wish to emphasize that these results are complementary to surface potential data derived from APBS, with more neutral complexes of G3 dendrimers interacting with each other, and complexes of G4 dendrimers (with more positive surface potential) showing only marginal to no interaction. Such preliminary data provide be a suitable starting point for future developments aimed at a thorough characterization of dendrimer–dendrimer interaction and cell internalization mechanisms.

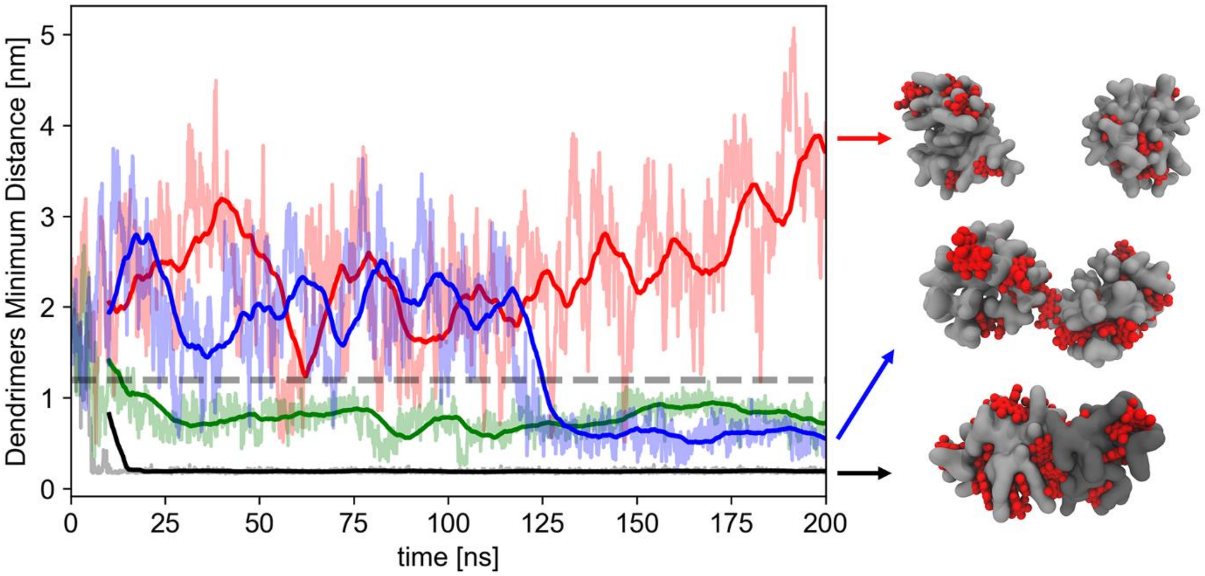

Figure S12. *Dendrimer–dendrimer minimum distance during the 200 ns MD simulation: G3 dendrimers interact after a few ns of simulation, whereas G4 dendrimers interact on longer time scales (PPI) or are not able to interact with each other (PAMAM). PAMAM G3 is presented in black, PAMAM G4 in red, PPI G3 in green, and PPI G4 in blue. Shaded colors show data from all processed trajectories, whereas solid colors represent moving averages. On the right, representative snapshots of the structural stability are presented for PAMAM G3, PPI G4, and PAMAM G4.*

Video S5, S6. *Representative trajectories of the 200 ns MD simulation for two-dendrimer systems involving PAMAM G3 and PPI G3 complexes, respectively, showing their ability to interact.*

Hydrodynamic diameter measurement

The solutions of complexes prepared as described in the point 4.2.2 of the Experimental Section were diluted in HEPES to a final dendrimer concentration of 10 µM and placed in the low volume sizing cuvettes (ZEN0112, Malvern Instruments Ltd., Malvern, UK). Measurements were performed at 25°C. The data were analyzed using the Malvern software. The particle size distribution was determined using a multimodal peak analysis, with individual peaks analyzed by number. Data were presented as mean ± SD, n = 3 (8 measurements each) (**Table S3**).

Table S3. *Hydrodynamic diameters of dendrimer:RB complexes. Data presented as average ± SD, n = 3 (8 measurements each).*

	Hydrodynamic diameter [nm]	Polydispersity index (PDI)
PAMAM G3:RB	232.52 ± 10.11	0.30 ± 0.06
PAMAM G4:RB	6.20 ± 2.42	0.28 ± 0.10
PPI G3:RB	311.50 ± 39.95	0.56 ± 0.18
PPI G4:RB	373.87 ± 59.28	0.60 ± 0.16

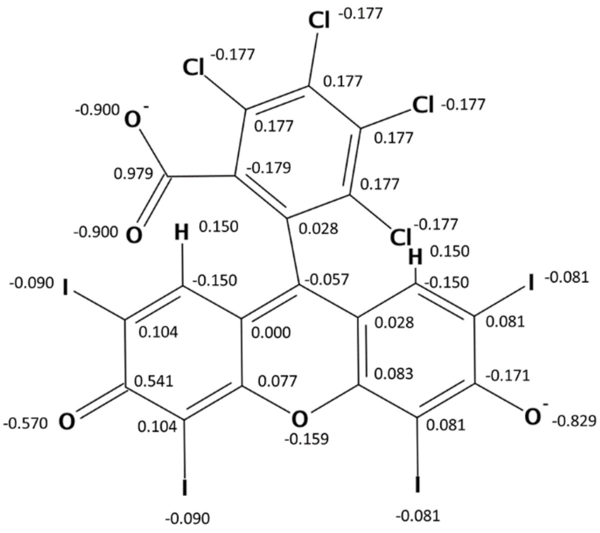

Figure S13. *Rose bengal (RB) structure with partial charges assigned using the AM1-BCC charge method.*

References

1. Maingi, V.; Jain, V.; Bharatam, P. V.; Maiti, P. K. Dendrimer Building Toolkit: Model Building and Characterization of Various Dendrimer Architectures. *J. Comput. Chem.* **2012**, *33* (25), 1997–2011.
2. Opitz, A. W.; Wagner, N. J. Structural Investigations of Poly(Amido Amine) Dendrimers in Methanol Using Molecular Dynamics. *J. Polym. Sci. Part B Polym. Phys.* **2006**, *44* (21), 3062–3077.
3. Barraza, L. F.; Zuñiga, M.; Alderete, J. B.; Arbeloa, E. M.; Jiménez, V. A. Effect of PH on Eosin Y/PAMAM Interactions Studied from Absorption Spectroscopy and Molecular Dynamics Simulations. *J. Lumin.* **2018**, *199*, 258–265.
4. Caballero, J.; Poblete, H.; Navarro, C.; Alzate-Morales, J. H. Association of Nicotinic Acid with a Poly(Amidoamine) Dendrimer Studied by Molecular Dynamics Simulations. *J. Mol. Graph. Model.* **2013**, *39*, 71–78.
5. Kanchi, S.; Gosika, M.; Ayappa, K. G.; Maiti, P. K. Dendrimer Interactions with Lipid Bilayer: Comparison of Force Field and Effect of Implicit vs Explicit Solvation. *J. Chem. Theory Comput.* **2018**, *14* (7), 3825–3839.
6. Kavyani, S.; Amjad-Iranagh, S.; Dadvar, M.; Modarress, H. Hybrid Dendrimers of PPI(Core)-PAMAM(Shell): A Molecular Dynamics Simulation Study. *J. Phys. Chem. B* **2016**, *120* (36), 9564–9575.
7. Ramos, M. C.; Horta, V. A. C.; Horta, B. A. C. Molecular Dynamics Simulations of PAMAM and PPI Dendrimers Using the GROMOS-Compatible 2016H66 Force Field. *J. Chem. Inf. Model.* **2019**, *59* (4), 1444–1457.
8. Lee, I.; Athey, B. D.; Wetzel, A. W.; Meixner, W.; Baker, J. R. Structural Molecular Dynamics Studies on Polyamidoamine Dendrimers for a Therapeutic Application: Effects of PH and Generation. *Macromolecules* **2002**, *35* (11), 4510–4520.
9. Porcar, L.; Liu, Y.; Verduzco, R.; Hone, K.; Butler, P. D.; Magid, L. J.; Smith, G. S.; Chen, W. R. Structural Investigation of PAMAM Dendrimers in Aqueous Solutions Using Small-Angle Neutron Scattering: Effect of Generation. *J. Phys. Chem. B* **2008**, *112* (47), 14772–14778.
10. Rathgeber, S.; Monkenbusch, M.; Kreitschmann, M.; Urban, V.; Brulet, A. Dynamics of Star-Burst Dendrimers in Solution in Relation to Their Structural Properties. *J. Chem. Phys.* **2002**, *117* (8), 4047–4062.
11. Liu, Y.; Bryantsev, V. S.; Diallo, M. S.; Goddard, W. A. PAMAM Dendrimers Undergo PH Responsive Conformational Changes Withoutswelling. *J. Am. Chem. Soc.* **2009**, *131* (8), 2798–2799.
12. Maiti, P. K.; Çağin, T.; Lin, S. T.; Goddard, W. A. Effect of Solvent and PH on the Structure of

PAMAM Dendrimers. *Macromolecules* **2005**, *38* (3), 979–991.
13. Topp, A.; Bauer, B. J.; Tomalia, D. A.; Amis, E. J. Effect of Solvent Quality on the Molecular Dimensions of PAMAM Dendrimers. *Macromolecules* **1999**, *32* (21), 7232–7237.
14. Scherrenber, R.; Coussens, B.; Van Vliet, P.; Edouard, G.; Brackman, J.; De Brabander, E.; Mortensen, K. The Molecular Characteristics of Poly(Propyleneimine) Dendrimers as Studied with Small-Angle Neutron Scattering, Viscosimetry, and Molecular Dynamics. *Macromolecules* **1998**, *31* (2), 456–461.
15. Prosa, T. J.; Bauer, B. J.; Amis, E. J.; Tomalia, D. A.; Scherrenberg, R. A SAXS Study of the Internal Structure of Dendritic Polymer Systems. *J. Polym. Sci. Part B Polym. Phys.* **1997**, *35* (17), 2913–2924.
16. Jain, V.; Maingi, V.; Maiti, P. K.; Bharatam, P. V. Molecular Dynamics Simulations of PPI Dendrimer-Drug Complexes. *Soft Matter* **2013**, *9* (28), 6482–6496.
17. Wu, C. PH Response of Conformation of Poly(Propylene Imine) Dendrimer in Water: A Molecular Simulation Study. *Mol. Simul.* **2010**, *36* (14), 1164–1172.
